# British Society for Matrix Biology Autumn 2020 Meeting: “Basement Membranes in Health and Disease”

**DOI:** 10.1111/iep.12381

**Published:** 2021-03-21

Report by

The 2020 BSMB Autumn meeting was planned as an in‐person get‐together of matrix biologists with a focus on the important role of basement membranes in health and disease. COVID‐19 put paid to the ‘in‐person’ part of proceedings, but thanks to the fantastic organizing committee of Dr. Tom Van Agtmael (Glasgow), Professor Rachel Lennon (Manchester) and Dr. Fabio Quandamatteo (Dublin), a three‐day online format was arranged. There were many interesting talks from both established researchers, up‐and‐coming young principal investigators, early career postdoctoral students and students. These results were disseminated to over 250 attendees around the world.

## Day 1

The webinar was opened by Florence Ruggiero (Lyon), Christos Kyprinanou (Cambridge) and Sally Horne‐Badovinac (Chicago). Florence has used the optical transparency of zebrafish embryos to demonstrate a role for collagen XV‐B in neuron axonal guidance during myofibre innervation. The Ruggiero laboratory findings suggest that tenascin‐C regulates deposition of collagen XV‐B, which in turn creates a less stiff microenvironment through which neural axons can migrate. This work is important as collagenopathies are associated with neuromuscular diseases, yet mechanisms as to how motor function is precluded in the myotome are lacking. Christos Kyprinanou gave an excellent presentation of his recent *Nature* article that outlined the importance of basement membranes in tissue sculpting, cell polarization and morphogenesis in pre‐ and postimplantation stage mouse embryos. Christos explained how Nodal, an important morphogen in early embryogenesis, stimulates MMP2 and MMP14 activity to create a thinner, and so weakened, basement membrane to permit mesendoderm ingression at the start of gastrulation. His work highlights the importance of the matrix in shaping tissues to enable proper embryogenesis. The final speaker of the first session was Sally Horne‐Badovinac, who showed unpublished data suggesting that stress fibres in the epithelial cells that surround the Drosophila egg chamber integrate into the basement membrane through focal adhesions that form at the front of the cell and then ‘treadmill’ towards the back before the adhesion is removed. This particular focal adhesion dynamic may help to polarize the basement membrane.

After a short break, where posters on matrix production in E. coli and the role of laminin subunit accumulation in a fruit fly model of Alzheimer’s disease were shown, the afternoon session was started by David Stephens (Bristol). Giantin is a large protein that is anchored to the cytoplasmic face of the Golgi and is likely to have a role in trafficking of matrix components to the extracellular space. In a CRISPR‐generated giantin knockout RPE1 cell line, David showed results from his laboratory that suggest cells lacking giantin are unable to process the N‐terminal propeptide from collagen, thereby leading to defects in polymerization in the extracellular space.

This talk was followed by five short presentations chosen from submitted abstracts. The exciting work shown included the first vertebrate tagging (with Dendra2) of a matrix molecule (Lamb1); a role for TTBK2 in regulating ADAMTS‐5‐mediated aggrecan degradation; an analysis of missense variants in *COL4A1* and *COL4A2* in patients with sporadic intracerebral haemorrhages; the use of atomic force microscopy to demonstrate the flexibility that interruption sequences provide type IV collagen; and an immuno‐matrix talk describing the recruitment of macrophages into kidney disease models. The penultimate talk of the day was given by Brian Stramer whose mathematical modelling and pulse‐chase experiments suggest a half‐life of type IV collagen of 12‐14 hours. Brian also used genetic deletions to show the importance of laminins and type IV collagen to ventral neural cord shape in Drosophila. The first day was completed by David Sherwood, who presented his laboratories latest insights into basement membrane‐to‐basement membrane fusions/linkages (B‐LINKs) and the role of hemicentin in anchoring two opposing basement membranes prior to their linkage.

## Day 2

The second day of talks commenced with Lydia Sorokin (Münster), who shared her groups’ research aimed at understanding the various mechanisms of leucocyte penetration of endothelial basement membranes. Lydia described how the interactions between endothelial laminins and distinct populations of integrin β1 receptors facilitate the pathogenicity and migration of leucocytes during the inflammatory response in a model of experimental autoimmune encephalomyelitis (EAE). This work will decipher the mechanisms involved in EAE disease progression.

In keeping with these sessions’ theme of understanding the role of basement membranes in various pathologies and diseases, we then heard five short presentations from selected abstracts. The first of which discussed work that utilized the benefits of *C. elegans* as an experimental model to identify potential genetic modifiers of collagen type IV disorders. Also focussing on collagen mutations and disease was an investigation into identifying the disease mechanisms of Vascular Ehlers Danlos syndrome. We also heard about work investigating the basement membrane‐associated protein Tinagl1 and how its disruption affects cilia‐dependant processes during embryogenesis in zebrafish. The molecular and skeletal characterizations of mice with fibrillin‐1 mutations was also presented, and the short‐talk session ended with a description of how peroxynitrite contributes to the phenotypic changes to vascular smooth muscle cells induced by extracellular matrix modification during atherosclerosis.

Following on from these talks, our next invited speaker Ambra Pozzi (Vanderbilt), introduced us to fused in sarcoma (FUS) and its relevance to kidney fibrosis. FUS nuclear translocation was found to regulate Collagen IV, shedding light on the mechanisms that control ECM synthesis and degradation. This research is important because it is an exciting insight into the molecular mechanisms underpinning pathological fibrosis, and identifies possible novel targets of pro‐fibrotic signalling.

After a short break and poster session, we then started the second session of the day with a focus on matrix secretion, assembly and turnover. Kevin Hamill (Liverpool) spoke to us about the exciting group of laminin derived protein fragments called netrins, specifically LaNt α31. Work is currently ongoing into characterizing the role of LaNt α31 in organogenesis and the mechanisms which are responsible for the defects observed when this netrin is mutated.

This session also featured five short talks from selected abstracts. Several of these talks described the hierarchical assembly and remodelling of basement membrane components during embryonic development. We were also shown how laminins are key regulators of cardiac growth and looping during early organogenesis, and the role of the basement membrane and the basal cells in the regulation of skin integrity in the zebrafish embryo. A model to investigate the role of perlecan in cardiac fibrosis using CRISPR/Cas9 edited human induced pluripotent stem cells was also presented. Finally, an investigation into how hyaluronan derived from the limbus regulates corneal lymphangiogenesis was described.

The day concluded with a final talk from Karl Kadler (Manchester). Karl explained the fascinating nature of collagen fibrils and the dynamic regulation of their secretion and turnover along with the elegant work that has determined the circadian control of procollagen type I fibrils. These studies have utilized several fluorescent tagging strategies such as Dendra2 and Nanoluciferase labelling in order to visualize the synthesis, transportation and secretion of collagen fibrils and how this is perturbed in tendon‐specific circadian clock knockout mice.

## Day 3

The final day of BSMB 2020 involved four invited speakers and three short presentations. The introduction to the session saw John Couchman (Chair of the BSMB) present the John Scott Young Investigator Award to Douglas Dyer (Manchester) for his work on glycosaminoglycans and their involvement in chemokine induced immune cell recruitment during inflammatory disease. Doug gave an excellent talk on his recent advances with a particular focus on CXCL4. Alex Nyström (Frieburg) presented his work on the discovery of potential therapeutic targets in epidermolysis bullosa, which is caused by mutations in *COL7A1*. Paul Potter (Oxford) presented his latest work on *Lama5* point mutations in a novel model of nephrotic syndrome and Keerthi Harikrishnan (South Carolina) presented her work on Fibulin‐1 and its role during aortic and pulmonary valve morphogenesis. Eileen Gentleman (Kings College) described her latest developments in TGF‐ß induced epithelial and matrix remodelling in gastrointestinal organoids and Taina Pihlajaniemi (Oulu) showed her groups, advances in collagen XVIII and their implications in homeostasis and pathology. Jeffrey Miner (St Louis) concluded the proceedings with his latest work on mutations in matrix genes including *COL4A5*, which are implicated in Alport syndrome. His work highlighted the exciting potential of a new exon skipping therapy.

The meeting close saw three awards for best short talks, sponsored by the International Journal of Experimental Pathology, awarded by the committee to Alaa Al‐Shaer (Vancouver) for her work on analysing the flexibility of collagen IV interruption sequences, Chris Derrick (Sheffield) for his talk on the regulation of cardiac growth by laminins and Christine Chew (Manchester) for her presentation on kidney macrophage heterogeneity and the contribution to matrix homeostasis.

The organizers did an outstanding job to deliver the meeting virtually without notable technical hitch. The meeting provided a prime opportunity to interact with the wider matrix community and engage with new research in the isolating times of the COVID‐19 pandemic. A virtual conference was an excellent alternative to a meeting that otherwise would have been postponed but we all look forward in hope of meeting in‐person next year at the University of Oxford for more captivating research into matrix biology.

**Richard Naylor**, **Erin Boland** **and** **Louise Hopkinson**

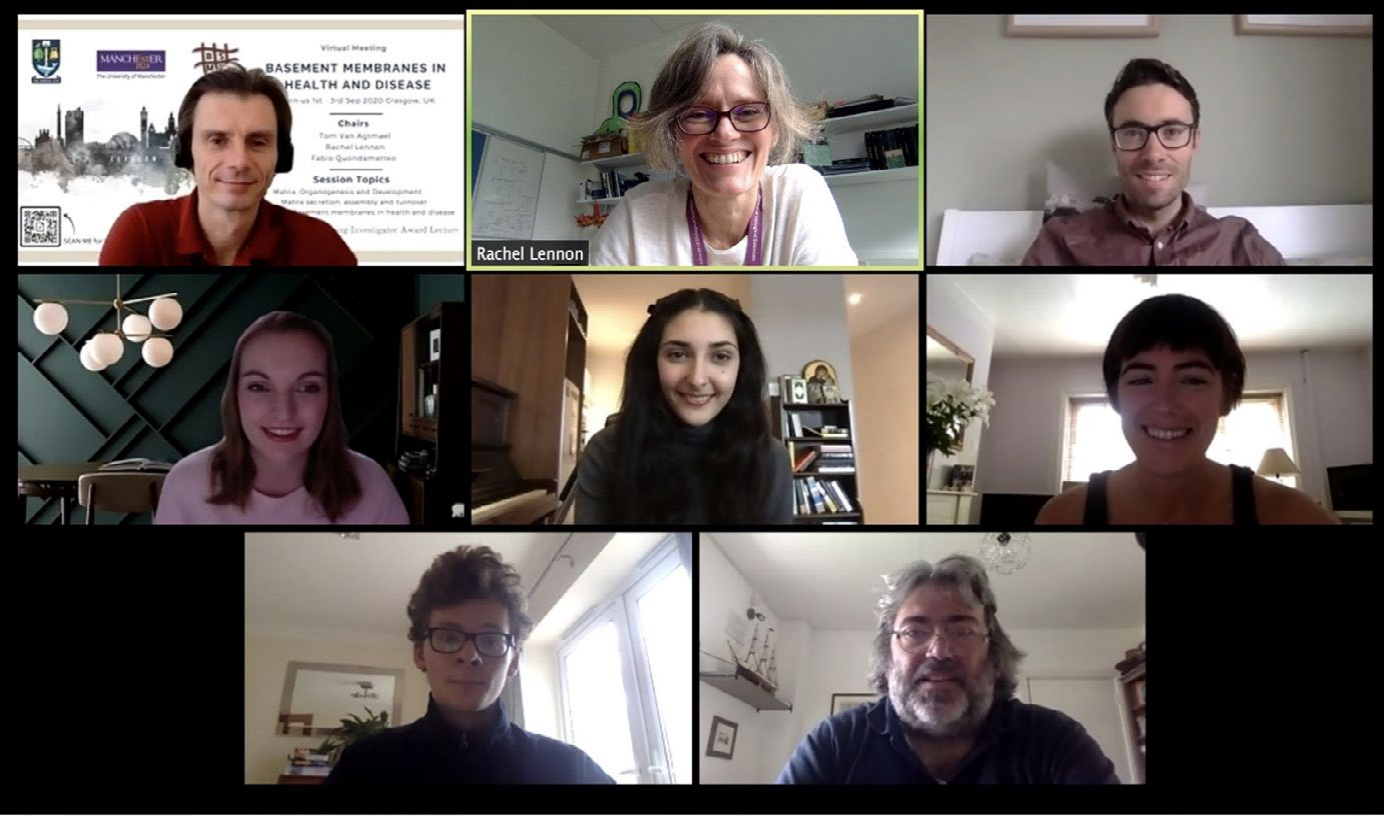

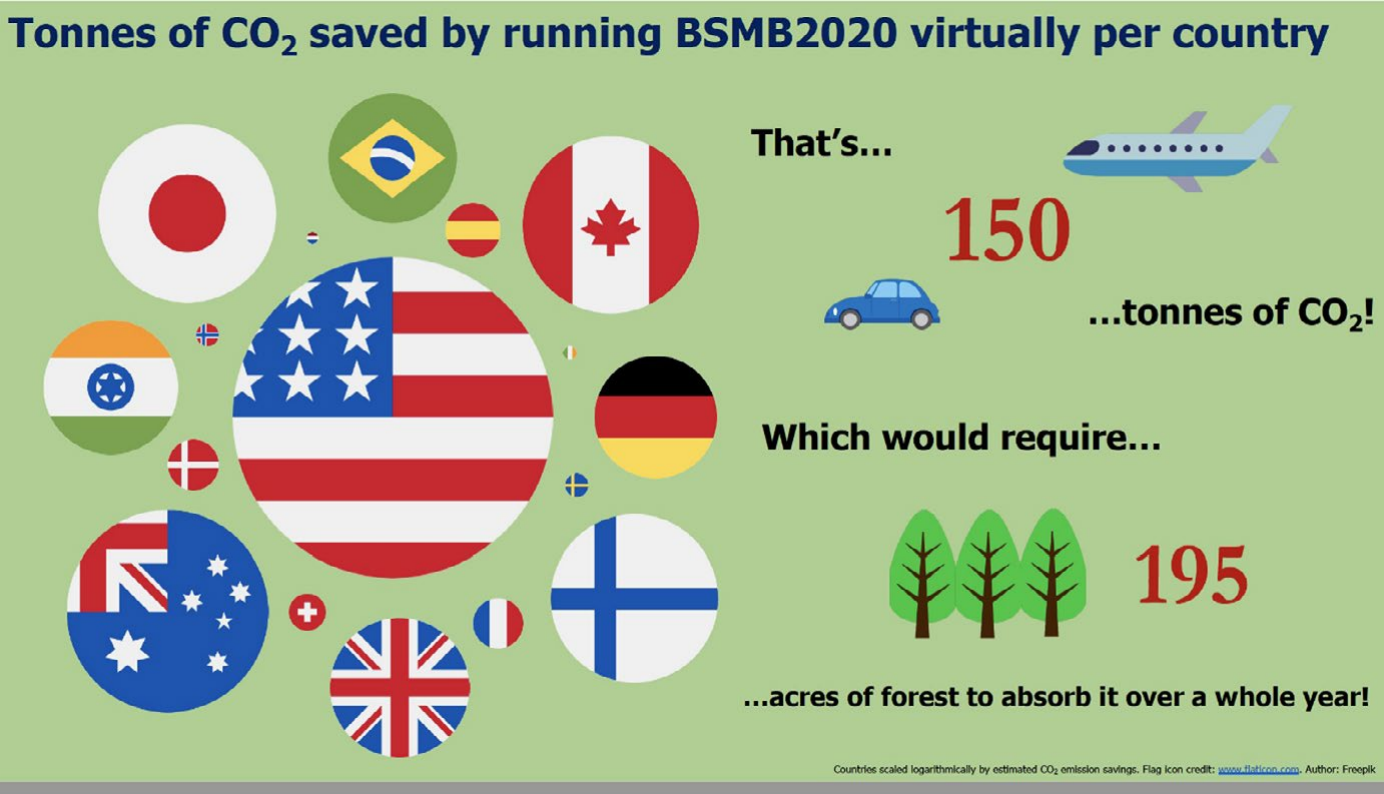

Photo by Rachel Lennon on the final day of meeting of the organizing team, from left to right, top to bottom: Tom Van Agtmael, Rachel Lennon, Richard Naylor, Louise Hopkinson, Malak Ammar, Anna Fildes, Adam Wolowczyk, Fabio Quondamatteo. Schematic by Adam Wolowczyk made to illustrate the CO2 savings thanks to the meeting being online.

## INVITED SPEAKERS ABSTRACTS

### Extracellular matrix and motor axon pathfinding: what zebrafish teach us

F. Ruggiero

*Institut de Génomique Fonctionnelle de Lyon, Ecole Normale Supérieure de Lyon, University of Lyon, 46 Allée d'Italie, 69364 Lyon, Cedex 07*

Despite the substantial advances in recent years, many challenges remain to understand the full complexity of the ECM functions in the formation and maintenance of the neuromuscular system. The role of ECM in motor axon guidance is one of them. As they exit from the spinal cord, growing motor axons reach their appropriate target muscles with a remarkable precision. Motor axons that travel a common route are bundled to form nerves and when a nerve is damaged, regrowing axons follow the same trajectory. This process relies on four major types of guidance cues: the diffusible and contact‐mediated molecules that can be either attractive or repulsive. Although the extracellular matrix (ECM) represents a major contributor of contact‐mediated cues by providing growing axons with local information, its composition, topology, cell source and specific functions are far from being fully elucidated. Using zebrafish, a popular model system widely used in basic and medical research, we found that developing muscle *spatially* and temporally shapes collagen‐containing matrices that are critical for motor axon pathfinding.

### Basement membrane remodelling regulates mouse embryogenesis

C. Kyprianou

*Mammalian Embryo and Stem Cell Group, Department of Physiology, Development and Neuroscience, University of Cambridge, Cambridge, UK*

The elongated early postimplantation mouse embryo (commonly referred to as the egg cylinder) is compartmentalized into the embryonic epiblast (giving rise to the embryo proper), the extraembryonic ectoderm (directly abutting the epiblast) and the visceral endoderm (a layer of cells lining both epiblast and extraembryonic ectoderm). A fourth component, however, in this elegant assembly of lineages, is a collagen‐ and laminin‐rich basement membrane (BM) that surrounds the embryo and located directly under the overlying visceral endoderm. We found that the BM provides the physical scaffold that shapes the egg cylinder while staying pliable and compliant to accommodate the growth of the embryo that it surrounds. We have discovered that the BM achieves this plasticity by active and widespread generation of multiple perforations in the embryonic compartment, attributed to the epiblast‐specific expression and activity of the collagenolytic matrix metalloproteinases MMP2 and MMP14. The resulting perforated morphology ensures controlled deformation of the BM in response to forces attributed to embryo growth while maintaining at the same time BM integrity. Corroborating this hypothesis comes from embryo growth defects upon pharmacological inhibition of matrix metalloproteinases. Furthermore, the MMP2 and MMP14 expression in the epiblast is driven by Nodal activity, which prior to anterior‐posterior axis establishment is ubiquitously active in the embryonic portion of the embryo. However, as anterior‐posterior axis is established through asymmetric inhibition of Nodal and localized activity in the future posterior of the epiblast, the distribution of perforations also becomes posteriorized to follow the pattern of Nodal activity. This redistribution radically changes the BM landscape creating a neatly defined posterior perforation domain that demarcates the position and extent of a yet to form primitive streak. Prepatterning the BM in anticipation to gastrulation proves to be instrumental in the controlled extension of the primitive streak and progression of gastrulation as elimination of these perforations leads to gastrulation failure in multiple aspects.

### Dynamic basement membrane remodelling for organ morphogenesis

S. Horne‐Badovinac

*Biophysical Sciences, University of Chicago, 929 East 57th Street, Chicago, IL 60637, USA*

Basement membranes are extensively remodelled during development to allow organ growth and to guide tissue morphogenesis. However, how this dynamic remodelling occurs is poorly understood. Working in the *Drosophila* egg chamber (developing egg), my laboratory previously showed that collective migration of follicular epithelial cells synergizes with new protein secretion to create a polarized network of fibrils within the existing, planar basement membrane. This polarized matrix then plays an instructive role in transforming the initially spherical egg chamber into an elongated egg. In this talk, I will highlight newer work from the laboratory exploring the distinct roles that Dystroglycan and integrins play in polarizing the egg chamber’s basement membrane.

### Intracellular trafficking and processing of procollagen

D. Stephens

*University of Bristol, Biomedical Sciences Building, University Walk, Clifton BS8 1TD, UK*

In animal models, knockout of the Golgi matrix protein giantin leads to skeletal and craniofacial defects. In our laboratory, we find that giantin mutant zebrafish accumulate multiple spontaneous fractures in their caudal fin, suggesting their bones may be more brittle. By experimentally inducing new fractures, we identified defects in the mineralization of newly deposited collagen at the site of injury as well as diminished procollagen reporter expression in mutant fish. To better understand these matrix deficiencies, we engineered giantin knockout human cells expressing a GFP‐tagged procollagen. While the trafficking of procollagen does not require giantin, we found that intracellular N‐propeptide processing of pro‐α1(I) is defective in these giantin knockout cells. These data demonstrate a conserved role for giantin in collagen biosynthesis and extracellular matrix assembly and provide evidence of a giantin‐dependent pathway for intracellular procollagen processing.

### Building an embryonic basement membrane from scratch

B. Stramer

*King’s College London, Great Maze Pond, London SE1 9RT, UK*

The basement membrane (BM) is a composite material that is generated by polymerization and complex interactions of numerous components. In order to understand how this network is built, we have been exploiting our unique capacity to live image the core BM components in *Drosophila* embryos at a stage of development when the animal is initiating production. We can therefore examine the timing of component expression and polymerization from nascent immature stages through to network homeostasis. I will show how our ability to examine BM initiation, when the network is far from equilibrium, has allowed us to elucidate novel dynamics involved in BM formation and function during animal development.

### Stably linking basement membranes—insights from *C. elegans*

C. A. Gianakas; D. Keeley; D. Sherwood

*Duke University, Department of Biology, Durham, NC 27708, USA*

Basement membranes (BMs) of adjacent tissues are often linked together to maintain long‐term adhesions. Long‐term BM‐BM adhesions occur in many vertebrate tissues such as the kidney, brain, eye and lung. Usually, these adhesions link a tissue to neighbouring vasculature allowing the exchange of nutrients or waste. Disruption of these adhesions can lead to pathologies such as Alport syndrome, in which genetic mutations in matrix components lead to glomerular BM splitting and kidney disfunction. Despite the importance of BM adhesions, very little is known about their regulation and maintenance. We are establishing a new model for investigating these adhesions—the basement membrane linkage (B‐LINK) in *C. elegans*. The B‐LINK is an adhesion complex that mediates BM‐BM adhesion between the BMs of the uterine and epidermal tissues and plays a critical role in supporting the uterine tissue during egg‐laying. Our current studies are identifying and characterizing new components of the B‐LINK. We have recently completed a visual screen of BM proteins to identify components that are enriched at the B‐LINK and paired this with an RNAi screen to determine those required for forming and maintaining the B‐LINK. To date, we have discovered four new B‐LINK matrix components (type IV collagen, fibulin, papilin and perlecan) and further characterized a previously identified B‐LINK component hemicentin. We have determined the functional role of these B‐LINK components to generate a working model of B‐LINK development. Broadly, we have found that these components are key regulators of BM adhesion, with hemicentin (and possibly fibulin) facilitating the initial BM‐BM adhesion upon which perlecan and type IV collagen (which requires papilin for its regulated deposition) are added to form the secure long‐term connection. This work aims to expand our knowledge of BMs and help us understand tissue adhesion in order to inform treatments for diseases caused by BM adhesion failure.

### Mechanisms of leukocyte penetration of endothelial basement membranes

L. Sorokin

*Institut für Physiologische Chemie und Pathobiochemie, University of Munster, Waldeyerstraße 15, D‐48149 Münster, Germany*

### What’s the ‘FUS’ about fibrosis

A. Pozzi

*Vanderbilt University, Medical Center North, Room/Suite B‐3115, Nashville, TN 37232, USA*

Our laboratory studies mechanisms of fibrosis, a disease characterized by excessive accumulation of extracellular matrix (ECM) components, which lead to altered tissue function and consequent loss of organ function. Although many factors contribute to pathological ECM synthesis, we study how cell‐ECM interaction via integrins regulates ECM production in healthy and diseased organs. Among the integrin family members, we focus on the collagen IV binding receptor integrin α1β1 and provide evidence that this receptor plays an anti‐fibrotic action. To this end, its genetic deletion leads to excessive accumulation of ECM and fibrosis following injury. One of our goals is to understand the mechanism(s) whereby this collagen IV receptor plays an anti‐fibrotic action in order to devise more selective therapies for the treatment of fibrosis. Using proteomic analysis, we found that a plausible mechanism is negative regulation of growth factor receptors, including the EGF receptor. Integrin α1β1‐mediated downregulation of EGF receptor signalling leads to reduced production of pro‐fibrotic reactive oxygen species, and reduced activation of transcription factors involved in ECM synthesis. As inhibition of EGF receptor and prevention of nuclear translation of these transcription factors ameliorate fibrotic response in mice lacking integrin α1β1, we suggest that targeting selective downstream signalling molecules regulated by this collagen IV receptor represents a promising approach for the treatment of fibrosis.

### LaNt α31, the ‘missing’ member of the laminin superfamily

K. J. Hamill

*Institute of Ageing and Chronic Disease, University of Liverpool, William Henry Duncan Building, 6 West Derby Street, Liverpool L7 8TX, UK*

Laminins and netrins are evolutionary‐related extracellular matrix proteins that together make up the laminin superfamily. The laminins are broken into α, β and γ chains with each laminin being an αβγ heterotrimer. The netrins, in contrast, contain only two groups; the γ laminin‐like netrins (netrin‐1, netrin‐3 and the GPI anchored netrin‐G1 and netrin‐G2) and the β laminin‐like netrin (netrin‐4). To this superfamily, we have added the ‘missing’ sixth group: the α‐laminin‐like netrins, which we have termed the laminin N‐terminus (LaNt) proteins. Unlike the netrins, the LaNt proteins are produced via intron‐retention and alternative polyadenylation from laminin encoding genes. Our work has focused on one member of this new class of protein, LaNt α31, derived from the LAMA3 gene. We have determined that LaNt α31 displays widespread expression at the transcript and protein level in intact tissue with notable enrichment throughout the vasculature, basal epithelium and in kidney tubules. Moreover, LaNt α31 upregulation was observed during wound reepithelialization, once limbal stem cells were activated, and was associated with breast ductal carcinoma and oral squamous cell carcinoma. Functional studies in corneal epithelial keratinocytes revealed that LaNt α31 co‐distributes and can form a complex with laminin 332, and that increasing LaNt α31 expression led to changes to laminin 332 organization, induced rapid assembly of hemidesmosomes and increased matrix metalloproteinase (MMP) activity. Moreover, induced LaNt α31 expression changed the mode of breast carcinoma cell invasion through a laminin‐rich matrix but not through collagen. The impact of LaNt α31 expression during development was investigated In vivo by generating a new transgenic mouse line where the UBC promoter drives LaNt α31 preceded by a flox‐stop cassette and crossing those animals with Rosa26‐Cre^ER2^ then using tamoxifen to drive Cre activation at embryonic day 15.5. The resulting offspring were born intact but not viable, presenting with localized erythema. Histological examination revealed extra‐vascular erythrocytes and widespread disorganization in multiple tissues consistent with basement membrane defects. In particular, the kidneys of the transgenic animals displayed epithelial detachment, tubular dilation, interstitial bleeding and thickening of the kidney tubule basement membranes. Changes were also apparent in the liver where a depletion in hematopoietic erythrocytic foci was identified. In the skin, disruption of the basal epithelial layers was evident in the interfollicular epithelium. Together, these findings point to the LaNt α31 protein, and by extension the other LaNt family members, as being capable of influencing important physiological processes, particularly during times of tissue remodelling. Importantly, the addition of a new class of protein to the laminin superfamily has potential to have wide‐ranging effects on many laminin‐mediated processes.

### Collagen fibril formation, the circadian clock and collagen reporters

K. E. Kadler; B. C. Calverley; J. Chang; R. Garva; A. Pickard; C.‐Y. C. Yeung

*Wellcome Centre for Cell‐Matrix Research, Faculty of Biology, Medicine & Health, University of Manchester, Manchester Academic Health Science Centre, Manchester M13 9PT, UK*

Collagen is the most abundant secreted protein in vertebrates and also one of the most long lived with a turnover half‐life exceeding hundreds of years ^1‐4^. The permanency of collagen contrasts with continued collagen synthesis by fibroblasts throughout adult life and with transcriptional/translational homeostatic mechanisms that replace damaged proteins with new copies. In a paper published earlier this year, we show circadian clock regulation of secretory pathway‐resident proteins that navigate procollagen‐I from its site of synthesis in the endoplasmic reticulum to secretion at the plasma membrane.^5^ The result is nocturnal procollagen‐I synthesis and daytime collagen fibril assembly, in mice. Rhythmic degradation maintains collagen homeostasis. This circadian cycle of collagen synthesis and degradation affects a pool of newly synthesized collagen‐I while maintaining the permanent collagen network. Disabling the circadian clock causes collagen accumulation. Thus, the circadian clock controls a sacrificial pool of collagen to maintain normal tissue function. In a recent study, we developed a new collagen reporter system, DyProQ^6^, which uses CRISPR‐Cas9 to introduce *nluc* (encoding NanoLuciferase, NLuc) into the *Col1a2* locus. This new method is upwards of 1000x more sensitive than hydroxyproline determination, maintains endogenous regulatory mechanisms and can be used in live cell imaging and multiwell formats. We demonstrated circadian clock regulation of collagen homeostasis, imaged procollagen‐containing transport vesicles in living cells, and screened a library of 1,971 FDA‐approved compounds in which we identified 10 candidates for repurposing in the treatment of fibrotic and 7 for degenerative diseases. The research was generously funded by Wellcome in the form of a Centre award (203128/Z/16/Z), a Senior Investigator Award (110126/Z/15/Z) and a 4‐year PhD studentship (210062/Z/17/Z). For correspondence, karl.kadler@manchester.ac.uk

1. Verzijl, N. *et al*. Effect of collagen turnover on the accumulation of advanced glycation end products. *J Biol Chem* **275**, 39027–39031, doi:https://doi.org/10.1074/jbc.M006700200 (2000).
2. Thorpe, C. T. *et al*. Aspartic acid racemization and collagen degradation markers reveal an accumulation of damage in tendon collagen that is enhanced with aging. *J Biol Chem* **285**, 15674–15681, doi:https://doi.org/10.1074/jbc.M109.077503 (2010).
3. Heinemeier, K. M., Schjerling, P., Heinemeier, J., Magnusson, S. P. & Kjaer, M. Lack of tissue renewal in human adult Achilles tendon is revealed by nuclear bomb (14)C. *FASEB J* **27**, 2074–2079, doi:https://doi.org/10.1096/fj.12‐225599 (2013).
4. Sivan, S. S. *et al*. Collagen turnover in normal and degenerate human intervertebral discs as determined by the racemization of aspartic acid. *J Biol Chem* **283**, 8796–8801, doi:https://doi.org/10.1074/jbc.M709885200 (2008).
5. Chang, J. *et al*. Circadian control of the secretory pathway maintains collagen homeostasis. *Nat Cell Biol* **22**, 74–86, doi:https://doi.org/10.1038/s41556‐019‐0441‐z (2020).
6. Calverley, B. C., Kadler, K. E. & Pickard, A. Dynamic protein quantitation (DyProQ) of procollagen‐I by CRISPR‐Cas9 NanoLuciferase tagging. *bioRxiv*, doi:https://doi.org/10.1101/2020.05.17.099119 (2020).

### From target identification to disease‐modifying therapies for a basement membrane disease

A. Nyström

*Department of Dermatology, Medical Faculty, Medical Center – University of Freiburg, Hauptstrasse 7, D‐79104, Germany*

In skin, collagen VII enables firm attachment of the epidermal basement membrane to the superficial papillary dermal extracellular matrix. Its deficiency causes the skin blistering disease dystrophic epidermolysis bullosa (DEB), which can be recessively or dominantly inherited. Secondary to skin blistering, DEB manifests with progressive, debilitating fibrosis, making DEB a model of tissue fragility‐driven fibrosis. The disease severity of DEB is on one hand determined by the abundance and residual functionality of mutated collagen VII and on the other one hand determined by mechanisms active during the natural progression of the disease. Starting from an increased understanding of disease mechanisms in recessive and dominant DEB, I will discuss the subsequent design and use of antisense oligonucleotides and natural peptides for disease‐modifying treatment of DEB.

### Towards a comprehensive view of the physiology and pathology of collagen 18

T. Pihlajaniemi

*University of Oulu, Pentti Kaiteran katu 1, Linnanmaa, Finland*

### Matrix, cells and kidney glomerular filtration

J. Miner

*Washington University School of Medicine, St. Louis, MO 63110, USA*

The glomerular basement membrane (GBM) is an integral component of the kidney’s filtration barrier that allows efficient crossing of water and small solutes but restricts the passage of plasma proteins greater than 60 kilodaltons. The GBM likely contains dozens of matrix proteins, but laminin‐521, type IV collagen, agrin and nidogens appear to be the major components. Hundreds of mutations in *LAMB2*, *COL4A3*, *COL4A4* and *COL4A5* have been reported to cause the human kidney diseases Pierson syndrome, Alport syndrome and thin basement membrane nephropathy. This demonstrates the importance of the laminin and collagen IV networks in establishing and maintaining the glomerular filtration barrier. We have investigated how the three primary glomerular cell types, the podocytes, endothelial cells and mesangial cells, respond to defects in the laminin and type IV collagen composition of the GBM. Our recent studies of podocytes, specialized epithelial cells that are critical for proper glomerular filtration, reveal that their actin cytoskeleton is specially tailored to withstand defects in the GBM, as well as to withstand toxic injury. These results suggest that the actin cytoskeleton could be a target for therapy in diseases that impact the podocyte.

## ORAL PRESENTATION ABSTRACTS

### Lamb1Dendra2—a new mouse model to study basement membrane dynamics in vivo

J. Morgner^*^; K. Hahn^*^; C. L. Iglesias^‡^; L. Kroese^†^; C. Pritchard^†^; P. Peters^‡^; I. Huijbers^†^; J. van Rheenen^*^

^*^Department of Molecular Pathology, Oncode Institute, Netherlands Cancer Institute, Amsterdam, The Netherlands; ^†^Mouse Clinic for Cancer and Aging, The Netherlands Cancer Institute, Amsterdam, The Netherlands; ^‡^The Maastricht Multimodal Molecular Imaging Institute, Maastricht University, Maastricht, The Netherlands

**Introduction:** The basement membrane (BM) maintains tissue architecture by separating epithelia and blood vessels from the surrounding tissue. Its function as a barrier is especially critical during tumorigenesis where it restricts malignant cell invasion into the stroma and intravasation into the vasculature in order to prevent metastasis formation. However, how BM formation and turnover are dynamically regulated and which cells types contribute to its remodelling is not understood. To study BM dynamics in vivo we developed a fluorescent BM mouse model based on the expression of a fusion protein of laminin beta1 (Lamb1) and the photoswitchable fluorophore Dendra2 that allows us to follow the dynamics of BM remodelling during homeostasis and tumorigenesis by using intravital microscopy (IVM).

**Materials and Methods:** Using the CRISPR/Cas technology, a cassette containing a flexible linker followed by the Dendra2 fluorophore and 3xFlag was inserted in‐frame into the last exon of Lamb1 achieving the expression of a Lamb1‐Dendra2‐3xFlag fusion protein in mice (Lamb1Dendra2). All organs were histopathologically analysed, Lamb1‐Dendra2 fluorescence expression was determined in a variety of organs, and the ultrastructure of the BM was examined by electron microscopy. Lamb1Dendra2 mice were combined with genetic models of breast cancer and organoid transplantation approaches were used to determine the source of BM production.

**Results:** Heterozygous and homozygous Lamb1‐Dendra2 mice express green fluorescent BMs in most tissues, including epithelia, endothelia and neuronal tissues. Histopathological and ultrastructural analysis revealed no differences compared to wild‐type animals. However, viable homozygous mice were strongly underrepresented (1/100), suggesting embryonic lethality in homozygous mice. Applying repetitive IVM through an imaging window to breast tumours in PyMT Lamb1Dendra2 mice, we follow BM formation and remodelling during tumour progression over multiple days to weeks. Using intravital time lapse imaging we determine how epithelial cancer cells and stromal cells behave in respect to BMs and how BM turnover is regulated by each cell type.

**Discussion:** With the development of this unique mouse model, we can answer fundamental questions of BM biology during tumorigenesis and homeostasis. For the first time, we look at the dynamics of mammalian structures that were previously assumed to be only static.

### Identification of a novel regulator of ADAMTS‐5‐mediated aggrecan degradation in chondrocytes

I. Collins ^*^; C. R. Coveney^*^; K. Yamamoto^†^; L. Troeberg^‡^; A. K. T. Wann^*^

^*^Centre for Osteoarthritis Pathogenesis Versus Arthritis, Kennedy Institute of Rheumatology, NDORMS, University of Oxford, Oxford, OX3 7FY, United Kingdom; ^†^Institute of Ageing and Chronic Disease, University of Liverpool, Liverpool, L7 8TX, United Kingdom; ^‡^Norwich Medical School, University of East Anglia, Norwich, NR4 7UQ, United Kingdom

**Introduction:** Disruption of extracellular matrix (ECM) homeostasis is linked to many diseases. For example, increased activity of matrix‐degrading proteases leads to cartilage loss in osteoarthritis (OA). However, therapeutic targeting of these proteases has so far been unsuccessful. To identify novel mechanisms regulating protease activity, our group has focused on an organelle called the primary cilium, as proteins involved in cilia assembly and function have been associated with matrix expression, organization and remodelling. We have seen that inducible, cartilage‐specific deletion of the ciliary protein IFT88 in mice results in tissue atrophy and OA, and that a hypomorphic IFT88 mutation results in elevated matrix catabolism in vitro, possibly due to disrupted regulation of protease activity by LRP‐1‐mediated endocytosis. Here, we aimed to identify other ciliary proteins that regulate proteolytic matrix degradation and determine the mechanism by which this occurs.

**Materials and Methods:** Proteins with different ciliary roles were depleted in a mouse chondrocyte line using siRNAs. Proteolytic matrix degradation was measured by culturing cells with purified aggrecan and subsequently detecting the aggrecan neoepitope AGEG by Western blot. qPCR and Western blotting were used to investigate mechanisms of protease activity regulation.

**Results:** Knockdown of the ciliary kinase TTBK2 resulted in increased aggrecan degradation, which was mainly mediated by the protease ADAMTS‐5. This increase was not associated with large increases in the expression of aggrecanolytic proteases, including ADAMTS‐5, or hedgehog signalling pathway targets. Levels of the endogenous protease inhibitor TIMP‐3 were unaffected by TTBK2 knockdown.

**Discussion:** These results indicate that TTBK2 is a novel regulator of ADAMTS‐5‐mediated aggrecan degradation. Experiments to determine how TTBK2 regulates ADAMTS‐5 activity, such as via ADAMTS‐5 endocytosis or activation, are currently underway, alongside analysis of the expression of *TTBK2* and other genes of interest in human OA and non‐OA cartilage, and ongoing investigations into the effect of ciliary gene knockdown on the extracellular matrix in vivo. Together, this work could potentially help to identify targets for restoring physiological levels of protease activity in OA, and provide further mechanistic insight into the regulation of matrix catabolism in cartilage and other tissues affected by diseases of pathological matrix degradation.

### Rare Missense Variants in COL4A1 and COL4A2 as a genetic risk factor in a Sporadic Intracerebral Haemorrhage patient cohort

G. Hamilton^*^; R. A.‐S. Salman^†^; I. Deary^‡^; T. Van Agtmael^§^

^*^Glasgow Polyomics, College of Medical, Veterinary & Life Sciences, University of Glasgow; ^†^Centre for Clinical Brain Sciences, Usher Institute of Population Health Sciences and Informatics, University of Edinburgh; ^‡^School of Philosophy, Psychology and Language Sciences, University of Edinburgh; ^§^Institute of Cardiovascular & Medical Sciences, College of Medical, Veterinary & Life Sciences, University of Glasgow

**Introduction:** Stroke is the second leading cause of death worldwide. Common genetic variants in *COL4A1* and *COL4A2* have been associated with intracerebral haemorrhage (ICH) in the general population while rare mutations, mostly affecting glycine resides, cause the hereditary COL4A1 syndrome. In addition to the common variants we hypothesized that rare coding variants in COL4A1 and COL4A2 could contribute to ICH in the general population.

**Materials and Methods:** We analysed sequencing data across 559Kbp at 13q34 including *COL4A1* and *COL4A2* among 1,492 individuals (192 ICH cases and 1300 controls) from a Scotland‐based cohort. The single nucleotide variants (SNVs) were annotated for minor allele frequency and functional impact using SnpEff, Ensembl’s Variant Effect Predictor (VEP) and gnomAD, the variants were further annotated with CADD scores from the Combined Annotation Dependant Database. The thermal stability of variants with the collagen triple helix was analysed using the Collagen Stability Calculator.

**Results:** Within the ICH cohort, we identified 4 rare missense variants and one that created a premature stop codon in *COL4A1*, variants were either not present in the control cohort or had a differing minor allele frequency. These rare variants are all classified as pathogenic by VEP and gnomAD and functionally described as deleterious. Additionally, the CADD scores for the rare variants were all greater than 24. Two of the missense variants, COL4A1^P890S^ and COL4A2^G1123E^, were located within the collagenous domain. Thermal stability analysis of these variants changed the melting temperature of the helix compared with the wild‐type protein.

**Discussion:** By performing the first large scale analysis of collagen IV in stroke, we uncovered that rare Collagen IV variants can occur in 3% of ICH in the general population. Our in silico analysis reveal the pathogenic nature of these variants, which will need to be verified. In contrast to COL4A1 syndrome, no variants affect the glycine residues highlighting a genotype‐phenotype correlation. However, two variants do effect the stability of the Collagen triple helix. These data highlight the important role of collagen IV in sporadic stroke, increasing our knowledge of its genetic basis.

### Sequence‐dependent flexibility mapping of collagen

A. Al‐Shaer ^*^; A. Lyons^†^; Y. Ishikawa^‡^; B. G. Hudson^§^; S. P. Boudko^§^; N. R. Forde^*,†^

^*^Department of Molecular Biology and Biochemistry and ^†^Department of Physics, Simon Fraser University, Burnaby, BC V5A 1S6, Canada; ^‡^Department of Ophthalmology, University of California San Francisco, School of Medicine, California, USA; ^§^Center for Matrix Biology, Vanderbilt University Medical Center, Nashville, TN 37232, USA

**Introduction:** Mechanics of extracellular matrices control diverse cellular functions, yet surprisingly little is known about the mechanics of the different collagens that comprise these structures. While the more abundant fibrillar collagens are best described as semi‐flexible polymers, much less is known about the flexibility of the network‐forming collagen type IV, an integral component of the basement membrane. A key feature that differentiates collagen IV from fibrillar collagens is the presence of interruptions of the triple‐helix‐defining (Gly‐X‐Y)_n_ sequence within the collagenous domain.

**Materials and Methods:** Here, we used atomic force microscopy to sample the two‐dimensional conformations adopted by collagen on mica and performed statistical analysis to calculate position‐dependent flexibility profiles.

**Results:** Our flexibility profile reveals that collagen IV possesses highly heterogeneous mechanics, ranging from semi‐flexible regions to a lengthy region of high flexibility towards the N‐terminus. A variable flexibility model based on distinct physical interruption classes fit the observed profile reasonably well, providing insight into the alignment of chains in collagen IV and supporting the role of interruptions in instilling flexibility. Limitations of this model were illuminated in an investigation of pN‐III collagen, which, although continuously triple helical, also exhibited variable flexibility along its length, notably possessing a high‐flexibility region around the matrix‐metalloproteinase binding site.

**Discussion:** As predicted for polymers of different rigidity subject to depletion interactions, the higher rigidity of the triple helix may drive more facile lateral collagen assembly into thick fibrils, while the comparatively flexible collagen IV is more limited in its assembly because of the higher entropic cost of packing its flexible domains into ordered structures. Studying different collagens offers the opportunity to examine the interplay between mechanical flexibility and higher‐order assembly, which may provide insight into their biological structures and functions.

### Kidney macrophage heterogeneity and contribution to matrix homeostasis

C. Chew ^*,†^; S. Rossi^*^; T. N. Shaw^*^; G. Howell^*^; S. Lui^*^; O. J. Brand^*^; C. Jagger^*^; R. Lennon^†^; T. Hussell^*^

^*^Lydia Becker Institute for Institute of Immunology and Inflammation, Manchester Collaborative Centre for Inflammatory Research (MCCIR), Manchester Academic Health Science Centre, The University of Manchester, Manchester, UK; ^†^Wellcome Centre for Cell‐Matrix Research, School of Biological Sciences, Faculty of Biology Medicine and Health, The University of Manchester, Manchester Academic Health Science Centre, Manchester, UK

**Introduction:** Glomerular disease is characterized by extracellular matrix deposition and the loss of filtration barrier integrity. The role of immune cells in glomerular disease is crucial for dampening inflammation and maintaining homeostasis. Macrophages are innate immune cells that respond to kidney inflammation; however, their role in matrix regulation is unknown. This project is aimed to characterize kidney macrophages and their interactions with the glomerular matrix.

**Materials and Methods:** Kidney immune cells were isolated from C57BL/6J, *Cx3cr1^CreER^* X *R26‐yfp* and *Ccr2^‐/‐^* mice and analysed using flow cytometry. Macrophage subsets were isolated by fluorescence‐ activated cell sorting and RNA extracted for bulk RNA‐sequencing. Matrisome‐associated secreted factors from cultured macrophage subsets were examined using a cytometric bead array.

**Results:** We found that macrophages can be defined phenotypically and transcriptionally into distinct subsets using the CX3C chemokine receptor 1 (CX3CR1) and a recently discovered resident macrophage candidate CD81 in the kidneys. These macrophage subsets were defined as CD45^+^Lineage^−^CX3CR1^−^CD81^−^ (P1), CX3CR1^−^CD81^+^ (P2) and CX3CR1^+^CD81^+^ (P3) cells. These macrophage subsets varied in their enrichment of matrisome‐associated secreted factors, including TNF‐α and IL‐10. Examining macrophage recruitment using tamoxifen‐treated *Cx3cr1^CreER^* X *R26‐yfp* mice, the P3 subset displayed the lowest rate of monocyte replenishment compared to P1 and P2 cells. In *Ccr2^‐/‐^* mice, a known model of defective bone marrow egress, P1 and P2 subsets were dependent on CCR2 in maintaining macrophage recruitment. Bulk RNA‐sequencing of isolated kidney macrophages revealed that these subsets had transcriptional profiles distinct from circulating blood monocytes. Interestingly, in gene ontology analyses applied to pairwise comparisons of differentially expressed genes between each population, many clusters were found with distinct transcriptional pathways of cell adhesion and extracellular matrix organization. Of the transcripts highlighted, these included *Col4a4*, *Col4a3* and *Col5a2* genes, with previously established roles in the glomerular basement membrane.

**Discussion:** Our data suggest that macrophages, in addition to their prevailing function within the immune system, are established in their diversity and may influence the matrix in kidneys. These novel findings suggest crucially, that macrophages may contribute to matrix organization, which may in turn shape the activation of immune responses.

### Identification of candidate suppressors of collagen type IV‐related disorders in the nematode *C. elegans*

A. Gatseva ^*^; I. Johnstone^†^; T. Page^‡^; T. van Agtmael^*^

^*^Institute of Cardiovascular and medical Sciences, University of Glasgow, Glasgow, G12 8QQ, United Kindom; ^†^School of Life Sciences, University of Glasgow, Glasgow, G12 8QQ, United Kindom; ^‡^Institute of Biodiversity Animal Health & Comparative Medicine, University of Glasgow, Glasgow, G61 1QH, United Kindom

**Introduction:** Identification of genetic modifiers of disease increases our understanding of pathomolecular mechanisms and holds the promise of finding novel therapeutic targets. Mutations in the extracellular matrix component collagen IV (Col4a1) cause cerebrovascular, eye and kidney disease, for which there are no treatments. *emb‐9(g23)* collagen mutant animals display temperature‐dependent embryonic lethality (at 20 ºC), matrix defects and intracellular collagen accumulation, reflecting the mammalian disease. Genetic suppressors can modulate Col4a1 disease outcome; however, their identity remains unknown. We employed a mutagenesis and whole‐genome sequencing (WGS) approach and compiled a list of 10 candidate suppressors of the mutant collagen phenotype at 20°C.

**Materials and Methods:** We employed a saturated EMS mutagenesis approach to induce random single nucleotide substitutions in the genome of *emb‐9(g23)* homozygous mutant strain. We examined the survival of newly generated strains at 20°C. We compiled a list of candidate suppressors, using WGS approach and comparative genomics analysis. RNAi‐mediated knockdown is used to determine the rescue potential of each candidate suppressor.

**Results:** We identified 40 suppressor populations, in which the embryonic lethality is rescued, displaying up to 70% increase in survival at 20ºC. We selected four strains for further investigation, displaying the largest increase in survival. Using a WGS approach, we have identified a candidate gene region in two suppressor strains. We identify novel EMS‐induced variants in suppressor strains within coding regions. Currently, employing RNAi‐mediated knockdown strategy to identify causal variant.

**Discussion:** Identifying genetic modulators of Col4a1 mutant phenotypes is important in revealing the molecular mechanisms of the disease and identifying novel therapeutic approaches. Rescuing the embryonic lethality of *emb‐9(g23)* to different extent suggests that we have generated a library of suppressors for Col4a1 disease in *C. elegans*. Identification of these genetic modifiers of collagen IV disease represents the first step in delineating their molecular mechanism, and their ‘translation’ to mammalian orthologs to determine if their efficacy is conserved in patient cell lines and mouse models of Col4a1 disease.

### Identifying disease mechanisms of vascular Ehlers Danlos Syndrome

R. Omar^*^; F. Malfait^†^; N. Bulleid^‡^; T. Van Agtmael^*^

^*^Institute of Cardiovascular and Medical Sciences, University of Glasgow; ^†^Centre for Medical Genetics, University of Ghent, Belgium; ^‡^Institute of Molecular Cell and Systems Biology, University of Glasgow

**Introduction:** Vascular Ehlers Danlos syndrome (vEDS) is a rare autosomal dominant disorder affecting connective tissues. It is the most serious form of EDS due to complications such as arterial and organ rupture that reduce life expectancy. vEDS is caused by mutations in collagen III, the majority of which affect glycine residues in the collagen Gly‐X‐Y repeat. The molecular disease mechanisms of vEDS remain unclear which hinders treatment development. To address this knowledge gap, we employed primary patient cell cultures to determine the mechanisms of COL3A1 mutations in vEDS.

**Materials and Methods:** We established and investigated primary patient fibroblast cells harbouring COL3A1 glycine mutations (G189S and G906R). Western blotting was used to assess collagen III secretion and ER stress. Sensitivity to trypsin digestion was used to determine the triple helical stability of secreted collagen III as a measure of collagen folding quality. Cells were treated with a chemical chaperone, PBA (4‐phenylbutyric acid), to investigate potential treatments.

**Results:** Both mutations caused intracellular collagen III retention, but this was more severe in COL3A1 G906R mutant cells. Increased sensitivity to trypsin digestion of secreted collagen III shows that the mutations allowed secretion of mutant misfolded collagen III. The intracellular accumulation of collagen III due to G906R cells caused activation of IRE1 and PERK ER stress pathways, with induction of ER stress induced apoptosis indicated by higher levels of CHOP. Interestingly, the G189S mutation resulted in an apparent milder level of ER stress without CHOP activation. We treated cells with the chemical chaperone PBA to determine its efficacy for COL3A1 mutations. A variety of PBA concentrations (5mM‐100uM) was able reduce ER stress in COL3A1 G189S cells, while only lower concentrations of PBA (100uM and 500um) reduced ER stress in COL3A1 G906R cells.

**Discussion:** We have identified that ER stress plays a role in the disease mechanism of vEDS, whereby individual COL3A1 mutations have differential activation of ER stress with higher ER stress levels being associated with more C‐terminal glycine mutations. Chemical chaperone treatment was successful in reducing ER stress levels. However, as COL3A1 mutations cause secretion of misfolded protein, it is now important to determine effects of PBA on matrix structure.

### Knockdown of basement membrane‐associated Tinagl1 disrupts cilia‐dependent processes in zebrafish embryogenesis

H. Neiswender; E. K. LeMosy

*Medical College of Georgia, Augusta University, Augusta, GA, USA*

**Introduction:** Tinag and Tinagl1 are found in basement membranes and some interstitial matrices. They share a structure—somatomedin B domain + inactive cathepsin B‐like domain—not seen in other proteins. Direct binding to laminin, integrins, Wnts and EGF‐R has been shown in vitro, and roles in EMT/MET, metastasis suppression and maintenance of differentiated epithelia proposed. Zebrafish have only a Tinagl1 ortholog, making it an attractive model for characterizing functions in vivo.

**Materials and Methods:** Zebrafish embryos were injected with splice‐blocking morpholinos or with Cas9 mRNA + a sgRNA targeting the first coding exon. In these animals, heart looping (2d), jaw cartilage (5d) and spinal cord organization and pronephric duct cilia (1d) were scored. In approaches disrupted by the pandemic, generating knockout and tagged lines is essential for confirming functional requirements and for identifying processes in which Tinagl1 participates.

**Results:** Knockdown of Tinagl1 expression using morpholinos (MOs), but not mismatch controls (MMs), demonstrated phenotypes similar to those documented for authentic cilia genes, for example disruption of L‐R asymmetry of heart looping, and pronephric dilatations. Cilia were shorter and fewer in number in the laterality organ and pronephric duct. G0 CRISPR/Cas9‐disrupted embryos showed similar results, supporting the possibility that these results are due to a real requirement for Tinagl1 in motile cilia homeostasis and/or function. This work is described in a BioRxiv preprint (June 2020) and is undergoing revision with a peer‐reviewed journal.

**Discussion:** Mutations in several proteins involved in matrix assembly, including laminin‐5, O‐xylosyltransferase and ADAMTS9, can result in polycystic kidney disease phenotypes in mice or human patients. For ADAMTS9, ciliogenesis of the primary cilium in renal epithelia is disrupted, while for the others, defects in basement membrane are proposed to result in cystogenesis. Relating ‘ciliopathy‐like’ phenotypes using zebrafish motile cilia to similar phenotypes in mammals involving cells bearing only primary cilia would require extensive experiments well beyond the current studies. This abstract is submitted in the spirit of opening conversation with basement membrane researchers.

### Molecular and skeletal characterization of mice with a fibrillin‐1 mutation: insight into tissue bioavailability of TGFβ in Marfan syndrome

G. Wilson; L. Pollock; E. Birch; I. Parisi; C. Duarte; B. Stott; J. M. Zarebska; C. Coveney; H. Muhammad; T. Vincent

*Kennedy Institute of Rheumatology, University of Oxford, Oxford, UK*

**Introduction:** Marfan syndrome (MFS) is caused by defects in the gene encoding the basement membrane protein fibrillin‐1 and results in aortopathy, skeletal abnormalities and dislocated eye lens. Pathogenesis is thought to involve dysregulated TGFβ activity although the precise nature of this is obscure. In this project we used mice with a human knock‐in mutation in fibrillin‐1 to characterize the skeletal manifestations of MFS in mice and to explore how and where latent TGFβ is sequestered in the tissue.

**Materials and Methods:** Mice containing the p.Cys1041Gly mutation were bred as heterozygotes (HET). Animals were culled at 9 and 45 weeks and tissues examined by microCT and immunohistochemistry (IHC) for skeletal proportions, kyphoscoliosis, growth plate anatomy and elastic lamina breaks within the aortic root. A new score for kyphoscoliosis was devised. Tissue sections of the aortic root and tibial growth plate were also examined by immunohistochemistry for latent TGFβ (LAP1) and perlecan.

**Results:** Mice with MFS exhibited all the skeletal features of the human condition including disproportionately extended long bones, kyphoscoliosis and rib cage abnormalities. There were increased elastic lamina breaks of the aorta of older mice compared with WT (>45 weeks). No significant difference in growth plate length or length of the hypertrophic region was observed in WT and HET mice, although the cell phenotype appeared qualitatively different. By IHC in WT mice, latent TGFβ co‐localized with perlecan in the endothelial basement membrane and tunica media of the aorta, lying adjacent to, and on either side of, the elastic fibres. In the WT growth plate latent TGFβ staining was largely restricted to the hypertrophic region.

**Discussion:** This study provides evidence that the murine model of MFS exhibits both the vascular and skeletal manifestations of the human condition. It suggests that latent TGFβ is sequestered in the matrix on perlecan within the aorta basement membrane and in a complex around elastin bundles of the tunica media and is localized predominantly in the hypertrophic region of the growth plate. It remains to be seen whether latent TGFβ localization is altered in disease and could explain the excessive growth and other skeletal manifestations of MFS.

### Phenotypic changes to vascular smooth muscle cells induced by extracellular matrix modification

S. M. Jørgensen; C. Y. Chuang; M. J. Davies

*Department Biomedical Sciences, Panum Institute, University of Copenhagen, Denmark*

**Introduction:** Vascular cell phenotype is controlled partly by the surrounding extracellular matrix (ECM). In vascular pathologies, including atherosclerosis, the ECM of the arterial wall is subject to modification by oxidants produced by inflammatory cells recruited to the vessel wall. The detection of extensive nitration on ECM components in atherosclerotic lesions has implicated peroxynitrous acid (ONOOH), a potent oxidizing and nitrating species, in lesion development. In the initiation and development of atherosclerosis, vascular smooth muscle cells (VSMCs) undergo a pronounced phenotypic switch from quiescent and contractile, to a proliferative and synthetic form. We hypothesize that ECM modification by ONOOH drives this phenotypic switch and thereby contributes to the progression of atherosclerosis.

**Materials and Methods:** Primary human coronary artery smooth muscle cells (HCASMCs)‐ECM were treated with various concentrations (1‐1000 µM) of ONOOH, and the extent of modification determined by ELISA. Cell adhesion to ONOOH‐modified ECM and subsequent proliferation were examined using calcein‐AM staining followed by the MTS assay after 48 hours and expression of mitosis‐related genes. Expression of various ECM and inflammatory genes was examined by qPCR.

**Results:** Extensive modification and nitration of ECM components was detected on HCASMC‐ECM treated with 10–1000 µM ONOOH. This was accompanied by reduced HCASMC adhesion to the modified matrix. Proliferation of HCASMCs on ECM modified by 10‐100 µM was increased and the expression of mitosis‐related genes was up regulated. Expression of other genes was also up regulated, including ECM proteins (laminin, fibronectin, and versican), inflammatory cytokines (IL‐1β and IL‐6), and vascular cell adhesion molecule (VCAM‐1) consistent with ECM remodelling and pro‐inflammatory state.

**Discussion:** This study provides a mechanism through which inflammation‐induced ECM modification may contribute to phenotypic switching of VSMCs, a key step in the formation of atherosclerotic lesions. Similar data have been reported recently for another oxidant, hypochlorous acid. These data highlight the potential of targeting oxidant formation in the prevention and treatment of atherosclerosis.

### Investigating basement membrane assembly and matrix remodelling during kidney development

M. R. P. T. Morais ^*,†^; P. S. Tian^*,†^; L. Hopkinson^*,†^; S. Kimber^†^; R. Lennon^*,†^

^*^Wellcome Centre for Cell‐Matrix Research; ^†^Faculty of Biology, Medicine and Health, University of Manchester, UK

**Introduction:** Kidney development involves many complex morphogenic events, some of which rely upon a temporal and spatial remodelling of the extracellular matrix and assembly of basement membranes (BM) that are critical for glomerular maturation and function. Since the composition and remodelling of kidney matrix, particularly of BM, during development is still not clearly understood, we sought to investigate BM assembly and remodelling in developing kidneys in vivo and in vitro.

**Methods:** We used high‐resolution label‐free mass spectrometry combined with a fractionation strategy for matrix enrichment to define the composition of BMs in the mouse kidney on embryonic day 19, and as a time‐course through iPSC‐derived kidney organoid differentiation.

**Results:** We identified over 200 matrix proteins in the mouse foetal kidney, which comprised 57 BM proteins that included well‐studied core components (type IV collagen, laminins, nidogen and heparan‐sulphate proteoglycans) plus minor proteins such as hemicentin, fibrillin and papilin, identified as key for BM growth and remodelling in other animal models. As expected, the matrix fraction obtained from the foetal kidney was enriched for BM (49% of total matrix abundance) whereas the cellular fraction presented high abundance of matrix‐associated proteins (64%). Collagen VI, fibronectin, nidogen and perlecan were the topmost abundant BM core components found in the matrix fraction, whereas the collagen‐associated chaperone serpin H1 showed the highest levels within the matrix‐associated category in both cellular and matrix fractions. Cross‐referencing of our foetal kidney proteomic data with human and mouse adult kidney datasets revealed a significant overlap particularly for BM proteins suggesting a conservation of BM composition from development to adulthood for these species, and identified new BM components found in the mouse foetal kidney such as thrombospondin‐4, spondin‐2 and pleiotrophin. With the kidney organoids, we identified 72 BM components in the iPSC‐derived kidney organoids, of which about 50 were also common to the mouse foetal kidney. Furthermore, the identification of BM proteins that undergo isoform switching during kidney development, increased significantly throughout the organoid differentiation time‐course, which is suggestive of BM remodelling and maturation for this in vitro system.

**Discussion:** In summary, the proteomic data in our study reflects the high complexity of the foetal kidney matrix at a protein level and will serve as a reference database for future studies aimed at investigating nephrogenesis.

### Laminins regulate cardiac growth through restricting second heart field addition

C. J. Derrick; F. Hussein; E. J. G. Pollitt; E. S. Noël

*Department of Biomedical Science, University of Sheffield, Firth Court, Western Bank, Sheffield, South Yorkshire, S10 2TN, United Kingdom*

**Introduction:** Congenital heart diseases occur in around 1% of live births and are structural malformations of the heart, arising through improper cardiac development. Heart looping morphogenesis is a critical stage in early vertebrate heart development when the heart transitions from a linear tube to an asymmetrically looped organ. Concomitantly, cells migrate into both cardiac poles from the Second Heart Field (SHF). These processes of morphogenesis and growth are intimately linked, and key for progressive heart development. During early development, the heart tube comprises an outer layer of contractile myocardium surrounding the endocardium, separated by cardiac extracellular matrix (ECM). We are interested in how interactions between these two tissue layers and the cardiac ECM drives heart morphogenesis.

**Materials and Methods:** Using a combination of transcriptomics and mRNA in situ hybridization, we identified dynamic and tissue‐specific expression of laminin subunit genes during early zebrafish heart morphogenesis. Subsequently, candidate genes were targeted for mutagenesis by CRISPR‐ Cas9. Heart morphology and SHF markers were analysed by mRNA in situ hybridization and SHF addition quantified using the transgenic lines *Tg(myl7:lifeActGFP); Tg(myl7:dsRed)*. To investigate interactions between heart function and SHF addition, we used an antisense morpholino oligonucleotide targeting *tnnt2a* to abrogate heart contractility.

**Results:** To examine laminin function in heart development, we generated *lamc1* (ortholog of human *LAMC1*) and *lamb1a* (ortholog of human *LAMB1*) zebrafish mutants. Loss of *lamc1* results in abnormal looping morphogenesis at 2dpf (days post fertilization) and increased heart size by 3dpf. Distinctly, loss of *lamb1a* also results in increased heart size by 3dpf, but does not impact early heart looping, highlighting that different laminin trimers perform distinct roles during cardiac development. *lamb1a* mutants have an increase in newly added SHF cells to the atrium at 2dpf, demonstrating that *lamb1a* limits SHF addition to the venous pole. Finally, knockdown of *tnnt2a* in *lamb1a* mutants rescues heart size at 3dpf, suggesting interactions between cell migration and biomechanics are regulated by *lamb1a* during heart development.

**Discussion:** Together, this work represents the first description of multiple roles for laminins in early vertebrate heart morphogenesis, reinforcing the importance of specialized ECM composition in driving distinct aspects of cardiac development.

### A CRISPR/Cas9 in vitro human‐induced pluripotent stem cell (hiPSC) model to investigate the role of perlecan in cardiovascular fibrotic disease

B. B. Johnson ^*^; T. L. Holmes^*^; J. L. Thompson^†^; M. Lord^‡^; C. Denning^†^; J. Whitelock^†,‡^; C. L. R. Merry^†^; James Smith^*^

^*^Norwich Medical School, University of East Anglia, Norwich Research Park, Norwich, NR4 7UQ, United Kingdom; ^†^Wolfson Centre for Stem Cells, Tissue Engineering and Modelling, University of Nottingham, Nottingham, NG72RD, United Kingdom; ^‡^Graduate School of Biomedical Engineering, University of New South Wales, Sydney, NSW 2052, Australia

**Introduction:** Cardiac fibrosis and heart remodelling are initially essential response processes following cardiac damage. However, prolonged progression can lead to heart failure, contributing to the biggest cause of death worldwide. Currently, there is no treatment for cardiac fibrosis, but heparan sulphate proteoglycans (HSPGs) are becoming targets of interest. Perlecan (HSPG2), is a basement membrane proteoglycan known for its binding to growth factors and extracellular matrix (ECM) components that become dysregulated in cardiac fibrosis. Perlecan has also been shown to alter calcium signalling and metabolism in other tissues, raising the possibility that it may perform similar roles during heart failure.

**Materials and Methods:** Human‐induced pluripotent stem cells (hiPSCs) were differentiated into cardiac fibroblasts and gene expression profiles were determined through qRT‐PCR during each major stage of the process: cardiac progenitor cell stage (CPC), epicardial progenitor cell stage (EPC) and cardiac fibroblast stage (CF). CFs were cultured with recombinant TGF‐β, and immunocytochemistry staining used to determine the formation of αSMA^+^ stress fibres. In parallel, the CRISPR/Cas9 system was used target perlecan in hiPSC lines.

**Results:** Expression analysis during CF differentiation showed the cardiac developmental genes TBX20 and GATA4 were upregulated in the CPC stage, indicating progression down a cardiac specific lineage. HSPG2 expression increased throughout the differentiation, leading to hiPSC‐CFs with an expression level similar to that of primary CFs. Activation of hiPSC‐CFs for 72 hours with 10ng/ml TGFβ induced the expression of αSMA^+^ stress fibres, indicating that a myofibroblast fibrotic phenotype could be modelled. Screening of the CRISPR/Cas9‐targeted hiPSCs identified heterozygous clones with reduced perlecan mRNA (~50% by qPCR), and protein expression by immunofluorescence.

**Discussion:** Our results show that we can successfully model cardiac fibrosis through the differentiation and activation of hiPSC‐CFs. Our CRISPR/Cas9 system successfully targeted the HSPG2 gene, creating clonal hiPSC lines with reduced expression. Our future work will use these perlecan targeted hiPSC lines within our model system to investigate the regulatory role of perlecan during cardiac fibrosis.

### Hyaluronan derived from the limbus is a key regulator of corneal lymphangiogenesis

M. Sun ^*^; S. Puri^*^; N. Mutoji^*^; Y. M. Coulson‐Thomas^†^; V. Hascall^‡^; D. G. Jackson^§^; T. F. Gesteira^*,†^; V. J. Coulson‐Thomas^*^

^*^College of Optometry, University of Houston, Houston, TX, USA; ^†^Universidade Federal de São Paulo, São Paulo, Brazil; ^‡^Cleveland Clinic, Cleveland, OH, USA; ^§^University of Oxford, Oxford, United Kingdom

**Introduction:** Corneal lymphangiogenesis and angiogenesis leads to the loss of corneal transparency. We have recently shown that in the cornea hyaluronan (HA) is present primarily in the limbal region. We investigated whether HA could play a role in regulating corneal lymphangiogenesis.

**Materials and Methods:** Wild‐type (wt) and hyaluronan synthase (HAS) knockout mice—specifically combined *Has1* ^‐/‐^ and *Has3* ^‐/‐^ null mice (*HAS1^‐/‐^;HAS3^‐/‐^*) and conditional *Has2* knockout mice (*HAS2^Δ/ΔCorEpi^*), were used. The mice were subjected to injury, alkali burn or suture placement, to investigate the role of HA on corneal lymphangiogenesis. Corneal buttons were also obtained from different developmental time‐points to study the role of HA in lymphatic vessel development. The corneas were analysed by whole mount immunohistochemistry and entire corneas were imaged under an LSM 800 confocal microscope using the both the z‐stack and tiling mode. Primary lymphatic vessel endothelial cells from human dermis (hDLECs) and lymph node (hLLECs) were used for tube formation assay and cell proliferation assay in vitro.

**Results:** After injury both wild‐type and *HAS1^‐/‐^;HAS3^‐/‐^* mice presented both an increase in HA expression and lymphangiogenesis. Interestingly, lymphatic vessels extended exclusively into HA rich areas. In stark contrast, *HAS2^Δ/ΔCorEpi^* mice did not upregulate HA synthesis after injury and, in turn, did not present lymphangiogenesis. Our developmental studies revealed first HA is expressed in the corneal limbus and thereafter lymphatic vessels invade this region. Our in vitro studies corroborated our in vivo data, with both HA increasing the proliferation and tube formation ability of hDLECs and hLLECs.

**Discussion:** HA regulates corneal lymphangiogenesis, both during development and after injury. These findings raise the possibility that therapeutic blockade of HA‐mediated lymphangiogenesis could be used to reduce corneal scarring and also prevent rejection after corneal transplantation.

### A novel model of nephrotic syndrome results from a point mutation in Lama5 and is modified by genetic background

S. Falcone^*,†^; T. Nicol^*,‡^; A. Blease^*^; M. J. Randles^§,¶^; E. Angus^**^; A. Page^**^; F. W. K. Tam^††^; C. D. Pusey^††^; R. Lennon^§^; P. K. Potter ^*,‡‡^

^*^MRC Harwell Institute, Mammalian Genetics Unit, Harwell Campus, Oxfordshire, United Kingdom; ^†^Centre for Cellular and Molecular Physiology, University of Oxford, Oxford, United Kingdom; ^‡^BHF Centre of Research Excellence, Division of Cardiovascular Medicine, Radcliffe Department of Medicine, John Radcliffe Hospital, University of Oxford, Oxford, United Kingdom; ^§^Wellcome Centre for Cell‐Matrix Research, The University of Manchester, United Kingdom; ^**^Biomedical Imaging Unit, Faculty of Medicine, University of Southampton, United Kingdom; ^††^Centre for Inflammatory Disease, Department of Immunology and Inflammation, Imperial College London, London, UK; ^‡‡^Department Biological and Medical Sciences, Faculty of Health and Life Sciences, Oxford Brookes University, United Kingdom

**Introduction:** The laminin α5 chain is essential for embryonic development and, in association with laminin β2 and laminin γ1, is a major component of the glomerular basement membrane (GBM). Variants in *LAMA5* were recently identified in children with nephrotic syndrome. We have identified a novel missense variant (E884G) of *Lama5* in the uncharacterized L4a domain of LAMA5 where homozygous mice develop massive proteinuria.

**Materials and Methods:** Mice were originally identified as from a phenotype‐driven screen of ENU mutagenized mice and subsequently backcrossed. Mice underwent biochemical analysis of plasma and urine. Histological and electron microscopy analyses were used to examine morphology. Assembly and export of mutant and wild‐type laminin heterotrimers were assessed by transfection and immunoblotting. Proteomic analysis of matrix was used to determine protein composition at different stages of disease.

**Results:** We characterized *Lama5* ^E884G/E884G^ mice that were identified as part of a phenotype‐driven screen to identify models of chronic and age‐related disease. Mutant LAMA5 was incorporated into the GBM, but proteomic and in vitro analyses suggest a reduced export of mutant protein and incorporation into the GBM. On two different genetic backgrounds (C3H and C57BL/6J), the phenotype was significant proteinuria, hyperlipidaemia and progressive chronic kidney disease. The proteinuria preceded a deterioration in kidney function and we observed podocyte effacement and invasion of the GBM. The progression of disease was slowed significantly on the C57BL/6J background, suggesting a modification of the response to the dysfunctional GBM.

**Discussion:** This novel model will provide insights into patho‐mechanisms of nephrotic syndrome, through a closer modelling of patient symptoms, and pathways that influence the response to a dysfunctional glomerular basement membrane. The results provide insight into how background genetic factor may affect the onset and severity of nephrotic syndrome’.

### Fibulin‐1 is critical for anterior, left coronary, non‐coronary leaflet formation during pulmonary and aortic valve morphogenesis.

K. Harikrishnan ^*,‡^; W. S. Argraves^*,†^

^*^Department of Regenerative Medicine and Cell Biology, Medical University of South Carolina, Charleston, SC, USA; ^‡^Biology Department, Indian Institute of Science Education and Research (IISER) Pune, Maharashtra, India; ^†^Passed away during the preparation of the manuscript.

**Introduction:** Fibulin‐1 (Fbln1), an extracellular matrix protein, is prominently expressed during outflow tract (OFT) morphogenesis as a component of both the endocardial cushions and OFT valves. Here, we sought to investigate the role of Fbln1 during semilunar valve formation.

**Materials and Methods:** WT and Fbln1‐deficient embryos were obtained from timed pregnant Fbln1 heterozygous females. AMIRA™ 5.3.3 was used to generate three‐dimensional (3D) images of the valves and expression of Fbln1. Cell counts were performed using Adobe Photoshop count tool. Immunolabeling and confocal microscopy was used to determine the TGFb2, Smad2/3 levels in the OFT. BrdU and TUNNEL staining was performed to evaluate proliferation and apoptosis.

**Results:** Morphometric analysis revealed that in Fbln1 null mouse embryos, the volume of the left, non‐coronary cusp of the aortic valve (AOV) and the anterior leaflet of the pulmonary valve (PV) were significantly reduced in size as compared to wild type (WT). In the left, non‐coronary cusp of the AOV and anterior leaflet of the PV, the size correlated with decreased cell numbers. Fbln1 null E14.5 embryos showed a decrease in cell numbers of both AOV and PV along with a localized region of apoptosis. Analysis of E12.5 hearts revealed a decrease in cell numbers accompanied by an increase in apoptosis with no change in the level of proliferation. Screening of genes that regulate apoptosis at E12.5 showed that both TGFβ2 and its signalling molecule pSmad2/3 levels were significantly increased in the OFT cushions of Fbln1 nulls. Furthermore, we also found Fbln1 expression to be highly restricted in the proximal OFT cushions at E12.5 in WT. Together, the findings indicate that Fbln1 is required to prevent hypoplastic semilunar valve formation.

**Discussion:** In this study, we have shown that Fbln1 is highly expressed by the proximal OFT cushions and is normally required to prevent excessive apoptosis. Our research findings are the first to identify a gene that is involved in the morphogenesis of specific semilunar valve leaflets highlighting the clinical relevance. This study has also identified Fbln1 as a novel candidate gene for pulmonary and AOV malformations.

### ILC1‐derived TGFβ1 drives epithelial and matrix remodelling in human iPSC‐derived intestinal organoids

G. M. Jowett^*,†^; M. D. A. Norman^*^; T. T. L. Yu^*^; P. Rosell^*,†^; E. Read^†^; J. F. Neves^†^; E. Gentleman^*^

^*^Centre for Craniofacial & Regenerative Biology, King’s College London; ^†^Centre for Host Microbiome Interactions, King’s College London

**Introduction:** Type‐1 innate lymphoid cells (ILC1) are enriched in patient mucosa with active inflammatory bowel disease (IBD), but the impact of this accumulation remains elusive, and α‐IFNy therapeutics against their signature cytokine lack clinical efficacy.

**Materials and Methods:** We established co‐cultures of murine small intestinal organoids (SIO) with ILC1, and human iPSC‐derived intestinal organoids (HIO) with patient ILC1. We also developed a functionalized, PEG‐based synthetic hydrogel system designed to form efficient networks at low polymer concentrations.

**Results:** Pico‐SMARTSeq2 transcriptomics on SIO co‐cultures revealed that IFNγ sensitizes epithelial cells to Fas‐mediated apoptosis. However, ILC1 also drive expansion of the epithelial stem cell crypt through p38γ phosphorylation and aberrant Cd44v6 expression, which is unexpectedly regulated by ILC1‐derived TGFβ1, not IFNγ. We next established that human ILC1 also secrete TGFβ1, and drive CD44v6 expression in both HIO epithelium and the surrounding mesenchyme, though notably this phenotype is only recapitulated by ILC1 from patient biopsies with active inflammation. As TGFβ1 is a master regulator of fibrosis, the leading indicator for surgery in IBD, we next characterized the ability of ILC1 to regulate matrix remodelling. We created modifiable PEG hydrogels that cross‐link quickly but at low stiffnesses and harnessed this platform to perform microrheology and atomic force microscopy on encapsulated HIO. We show that ILC1 drive matrix stiffening and degradation, which we posit occurs through a balance of MMP9 degradation and TGFβ1‐induced fibronectin deposition.

**Discussion:** Our synthetic organoid co‐culture system enabled us to tease apart an important role for intestinal ILC1 in epithelial and matrix remodelling, which may drive either wound healing or fibrotic pathologies in IBD. Moreover, this controlled 3D microenvironment provides a broader platform for dissecting interactions between complex hiPSC‐derived tissues and rare cell subtypes in development and disease.

## POSTER ABSTRACTS

### Production of soluble basement membrane proteins in the cytoplasm of E. coli

A. A. Sohail; M. Gaikwad; L. W. Ruddock

*University of Oulu, Finland*

**Introduction:** Basement membranes (BM) are thin layers of extracellular matrix deposition which surrounds cells of epithelial, muscle, adipose and the endothelium lining. Proteins which are major components of BM are perlecan, agrin, collagen IV, laminins and nidogens. These proteins are rich in disulphide bonds, which are essential for protein’s structure and function. Unfortunately, very limited atomic structures of BM proteins are available due to low protein yield. Our group has created a technology, CyDisCo, which helps *E. coli* to produce disulphide‐rich proteins including BM proteins in their native folding state with good yields.

**Materials and Methods:** Synthetic genes were used to produce BM protein fragments using PCR amplification. Expression of fragments is optimized using different variables, for example competent cells, culture media, temperatures and CyDisCo variants. Optimal conditions were carried out to a scale to allow multi‐milligram yields. Protein purification was initiated with immobilized metal‐affinity chromatography, followed by ion exchange chromatography and finally with size exclusive chromatography. Quality of the fragments is analysed using SDS‐PAGE, mass spectrophotometer (MS), circular dichroism (CD) and thermofluor.

**Results:** Multiple BM protein fragments have been produced and analysed using CyDisCo. For example, mouse perlecan fragment (G503‐T1672) which has 1169 amino acids and is thought to contain 44 disulphide bonds was successfully produced in a soluble state, with a purified yield of 4.4 mg/L. A single band on non‐reducing SDS‐PAGE suggested a single redox state and MS analysis verified that it has the expected molecular weight with all the cysteines in disulphide bonds. Information from CD and thermofluor analysis confirmed that the protein is in folded state and is thermally stable.

**Discussion:** Successful production of a large disulphide‐rich BM protein fragment in *E. coli* is a remarkable achievement. This encourages us to test other BM proteins and their complexes. The group is also developing a second generation of CyDisCo technology, which might help in producing full length BM proteins in cytoplasm of *E. coli*.

### Aβ toxicity rescued by ER retention of laminin monomers

J. H. Catterson ^*,†,1^; L. Minkley^*^; S. Aspe^*^; S. Judd‐Mole^*^; A. S. Moura^*^; M. C. Dyson^*^; A. Rajasingam^*^; N. S. Woodling^*^; M. L. Atilano^*^; M. Ahmad^*^; T. L. Spires‐Jones^†^; L Partridge^*,‡^

^*^Institute of Healthy Ageing, Genetics, Evolution and Environment, University College London, Darwin Building, Gower Street, London WC1E 6BT, United Kingdom; ^†^Centre for Discovery Brain Sciences, UK Dementia Research Institute, The University of Edinburgh, 1 George Square, Edinburgh, EH8 9JZ, Scotland, United Kingdom; ^‡^Max Planck Institute for Biology of Ageing, Joseph‐Stelzmann‐Strasse 9b, 50931 Cologne, Germany; ^1^Present address: Centre for Discovery Brain Sciences, UK Dementia Research Institute, The University of Edinburgh, 1 George Square, Edinburgh, EH8 9JZ, Scotland, United Kingdom

**Introduction:** Accumulation of Aβ in the brain is one of the hallmarks of Alzheimer’s disease (AD). In the adult *Drosophila* brain, human Aβ overexpression is toxic and leads to deterioration of climbing ability and shortened lifespan. However, it remains unknown if Aβ is inherently toxic or if it triggers toxic downstream pathways that lead to neurodegeneration.

**Materials and Methods:** We used a drug‐inducible *Drosophila* AD model that expressed the highly aggregative Arctic Aβ_42_ (Aβ^Arc^), which when expressed in the neurons of adult flies shortens lifespan and induces behavioural defects and neurodegeneration. We used the short‐lifespan phenotype of this model to screen for modifiers of Aβ toxicity. For verified hits, we then did further analysis including measuring effects of the modifier on Aβ levels ‐ including qPCR and ELISA. Immunohistochemistry was used to measure secretion of Aβ, and to identify cellular localization of proteins of interest.

**Results:** We identified the extracellular matrix protein subunit Laminin B1 (LanB1) as a robust modifier of Aβ toxicity. Despite high Aβ levels, LanB1 overexpression resulted in a robust rescue of toxicity, highlighting a potential protective mechanism of resistance to Aβ. Overexpression of other Laminin subunits and a Collagen IV subunit also significantly rescued Aβ toxicity, while combining LanB1 with these subunits led to an even larger rescue of toxicity. Imaging revealed retention of LanB1 in the ER, while LanB1 had no effect on the secretion of Aβ into the extracellular milieu. LanB1 also rescued toxicity independently of the IRE1α/XBP1‐mediated branch of the ER stress response. Interestingly, overexpression of ER‐targeted GFP also rescued Aβ toxicity indicating a broader benefit of ER protein retention in the context of neuronal Aβ.

**Discussion:** Typically, retention of laminins in the ER is detrimental to cellular health, but in the context of neuronal Aβ toxicity it may prove to be beneficial, and could be a new therapeutic avenue for AD.

### von Willebrand factor D domains production in cytoplasm of *E. coli*

M. Gaikwad; A. A. Sohail; L. W. Ruddock

*University of Oulu, Finland*

**Introduction:** Extracellular matrix (ECM) is a complex system made up various large proteins. Mucin 2 and Alpha tectorin are examples of ECM proteins. These seemingly unrelated proteins contain homologous domains called the von Willebrand D (VWD) domains. These VWD domains are indicated to play important roles in the folding and formation of mature proteins. Mutations in these proteins often lead to disease states. Due to their high disulphide bond density and complex nature, these proteins are hard to produce in non‐mammalian systems. We have recently expressed some VWD domain containing proteins in *E. coli* cells with the help of the CyDisCo system developed by our group.

**Materials and Methods:** Codon optimized synthetic genes were used as templates for PCR amplification of the target protein constructs. Expression test were carried out for each construct by using different expression strains, media and temperature. The best results were used for large scale production. The purification of these constructs was carried out in a 3‐step process which included IMAC, anion exchange and gel filtration respectively. The purified constructs were analysed using mass spectrometry, circular dichroism and thermofluor.

**Results:** VWF‐D domains from mucin 2 and alpha tectorin were successfully produced. The alpha tectorin construct contains 283 amino acids (8 disulphide bonds) while mucin 2 region contains 362 amino acids (15 disulphide bonds). Both the constructs were successfully purified with yields of 5.5 mg/L and 6.5 mg/L respectively. Their CD analysis were consistent with the published structures of the homologous domains. Thermoflour analysis showed the constructs to be very thermostable.

**Discussion:** As per our knowledge, this is the first‐time disulphide‐rich human von Willebrand factor D domains have been successfully expressed in *E. coli* cells. This opens a whole new avenue where different ECM proteins can be tested and expressed in *E. coli* systems. This could make it easier to study the protein that have till now been under studied.

### Investigating glycosaminoglycans in development using human stem cells and fully defined 3D cell culture environments

J. L. Thompson ^*,†^; S. Pijuan‐Galitó^*,†^; J. C. Ashworth^*^; Z. Nizamudeen^*^; J. Boveé^‡^; J. Smith^§^; A. Hook^†^; K. P Arkill^*^; M. R. Alexander^†^; L. Kjellén^¶^; C. L. R. Merry^*^

^*^Biodiscovery Institute and ^†^School of Pharmacy, University of Nottingham, United Kingdom; ^‡^LUMC, Netherlands; ^§^Norwich Medical School, University of East Anglia, United Kingdom; ^¶^Department of Medical Biochemistry and Microbiology, Uppsala University, Sweden

**Introduction:** Mutations in EXT1 and EXT2 heparan‐sulphate (HS) co‐polymerases disrupt normal glycosaminoglycan (GAG) production and GAG‐related signalling. In mice, EXT mutations result in embryonic lethality. In humans, EXT mutations are associated with the rare developmental disease Multiple Osteochondroma (MO). We have combined human induced pluripotent stem cells (hiPSC) and a 3D culture environment to generate a defined, GAG‐free MO disease model.

**Materials and Methods:** hiPSCs (EXT1+/‐, EXT2‐/‐) were created by reprogramming MO patient fibroblasts and by CRISPR/Cas9 gene editing of wild‐type hiPSCs. Cell behaviour and GAG composition was assessed in standard 2D culture and during 3D culture in a defined peptide hydrogel. Gel‐encapsulated cell clusters were high‐pressure frozen and transferred to cryo‐OrbiSIMS for high‐resolution mass spectrometry imaging of 3D deposited GAG.

**Results:** As expected, we were not able to detect any HS in EXT2‐/‐ hiPSCs. However, we did see differences in chondroitin sulphate/dermatan sulphate composition and, interestingly, observed an increase in levels of a non‐HS/CS/DS sulphated GAG species. Contrary to studies using HS‐deficient mouse embryonic stem cells, EXT2‐/‐ hiPSCs could signal via FGF2, and were able to differentiate to neural precursors.

**Discussion:** These data highlight differences in the roles of HS in mouse and human development, strengthening the need to develop robust in vitro human models. We demonstrate generation of novel cell tools and a GAG‐free 3D biomaterial, and combining these has allowed detailed analysis linking GAG composition and location. This will broaden our understanding of cell‐matrix interactions, and begin to unravel complex GAG structure‐function relationships.

### Studying the effect of osteoarthritis‐associated DNA methylation changes in transcriptional enhancer regions on chondrogenic gene expression

M. J. Burgers ^*^; K. Cheung^†^; D. A. Young^*^; L. N. Reynard^*^

^*^Skeletal Research Group, Newcastle University Biosciences Institute; ^†^Bioinformatics Support Unit, Faculty of Medical Sciences, Newcastle University

**Introduction:** Osteoarthritis (OA) is a degenerative joint disease and the leading cause of global disability in people over the age of 50. OA is characterized by cartilage extracellular matrix degradation and has been associated with aberrant cartilage DNA methylation. A genome‐wide DNA methylation meta‐analysis performed in our laboratory identified osteoarthritis‐associated DNA methylation changes, which were depleted in gene promoters and highly enriched in putative transcriptional enhancer regions. We therefore aimed to investigate the effect of these enhancer DNA methylation changes on gene expression and disease.

**Materials and Methods:** Fourteen regions with OA‐associated DNA methylation changes and an active enhancer signature in cartilage based on histone marks and open chromatin were selected. The chondrosarcoma cell line SW1353 was used to study the effect of DNA methylation in vitro. Enhancer activity and the effect of in vitro DNA methylation of these regions in SW1353 cells was assessed by luciferase assays, in which the enhancer was cloned in the CpG‐free pCpGL‐EF1 vector. SW1353 cells were treated with DNA hypomethylating agent 5‐Aza‐2′‐deoxycytidine (5‐Aza, 0.25 µM, 72 h). Gene expression and chromatin accessibility changes were assessed by RNA‐seq and ATAC‐seq respectively.

**Results:** The selected enhancer regions exhibited up to eleven fold higher luciferase activity than control, but enhancer activity was strongly reduced upon in vitro DNA methylation. DNA methylation was reduced up to 30% upon 5‐Aza treatment, although the level of this loss was variable between sites. Altered chromatin accessibility was identified in 29,401 regions, of which 73.7% had decreased accessibility, including 5 of the 14 OA‐associated enhancer regions studied. A total of 1382 and 1420 genes were significantly 1.5 fold up‐ and downregulated, respectively, after 5‐Aza treatment. Together these data indicate that loss of DNA methylation can have opposing effects on both enhancer chromatin accessibility, enhancer activity and putative target gene expression, with the effect of methylation being context dependent.

**Discussion:** OA‐associated DNA methylation changes are enriched in cartilage enhancer regions, with hypomethylation associated with changes in enhancer chromatin accessibility, activity and target gene expression. Understanding how enhancer DNA methylation regulates chondrogenic gene expression will give more insight in osteoarthritis progression and possible treatment.

### Short term exposure to physiologically relevant levels of interleukin 1β initiate sustained changes in chondrocyte

R. Horne ^*^; G. Bou‐Gharios^*^; D. Young^†^; S. Tew^*^

^*^Department of Musculoskeletal Biology, Institute of Ageing and Chronic Disease, University of Liverpool; ^†^Skeletal Research Group, Institute of Genetic Medicine, Newcastle University

**Introduction:** Inflammatory cytokines such as interleukin 1β (IL‐1β) are present within the synovial joint at low concentrations and demonstrate a slight elevation in response to mechanical stress and joint diseases such as osteoarthritis (OA). Although elevated in disease, these well‐studied factors do not reach the levels routinely used in experimental model systems. Our understanding of cartilage responses to physiologically relevant levels of inflammatory factors is therefore more limited. In this study, we used the secretion of translationally regulated cytokines to assay the early response of chondrocyte cells to physiologically relevant levels of inflammatory stimulation.

**Materials and Methods:** SW1353 chondrosarcoma cells and human articular chondrocytes (HACs) collected from patients undergoing total knee replacement were cultured as a model of chondrocyte function. Cells were stimulated with either high (10 ng/ml) or physiologically relevant (10 pg/ml) levels of IL‐1β and downstream cytokine secretion and activation of signalling pathways were characterized by ELISA and Western blotting.

**Results:** Dose‐dependent ELISA experiments demonstrate that SW1353 and HACs show a similar pattern of IL‐6 secretion in response to IL‐1β stimulation, with a dynamic range of minimal to maximal secretion ranging from 1 pg/ml to 100 pg/ml. Western blot analysis showed that stimulation of SW1353 cells with high dose (10 ng/ml) of IL‐1β activates MAPK signalling pathways very rapidly. A lower, physiologically relevant cytokine dose (10 pg/ml) resulted in significantly reduced MAPK signalling, despite causing 50% of maximal IL‐6 secretion, indicating that there is a distinct signalling environment in comparison to that produced by the high levels commonly used experimentally. Finally, pulsed exposure of cells to IL‐1β showed that stimulation for at least the first three hours of a 24‐h period with low levels of IL‐1β results in the same total secretion of IL‐6 as that caused by constant exposure.

**Discussion:** Measuring the secretion of translationally regulated cytokines such as IL‐6 has provided a novel output for how physiological levels of a cytokine influence chondrocyte function. Using this approach, we have demonstrated that chondrocytic cells require low, physiologically relevant levels of IL‐1β to drive IL‐6 secretion. Interestingly, the level of IL‐1β required to drive 50% secretion resulted in very low levels of MAPK signalling compared to that driven by commonly used experimental concentrations. Whether IL‐6 secretion levels are controlled by altered signalling levels or via discrete signalling systems needs to be determined. Finally, we found that chondrocytic cells continue to exhibit a response to the IL‐1β stimulus following its removal, with the cells primed to stimulate maximal levels of IL‐6 over 24 hours, after only 1‐3 hours of initial stimulation. Our findings suggest that physiologically relevant cytokine stimulation can rapidly initiate a sustained change in chondrocyte secretory profiles Future work will examine the signalling process involved in physiologically relevant cytokine responses, the role of protein translation regulation, and the effects that secondary cytokine release have on chondrocyte function.

### In a surgical model of OA female mice compared to males display pain at an early stage of disease and differential regulation of pain sensitizers

V. Batchelor ^*^; I. S. von Loga^*^; C. Driscoll^*^; A. Burleigh^*^; S.‐L. Chia^*^; B. Stott^*^, J. Miotla‐Zarebska^*^; D. Riley^*^; F. Dell’Accio^†^; T. L. Vincent^*^

^*^Kennedy Institute of Rheumatology, University of Oxford; ^†^William Harvey Institute, Queen Mary University of London

**Introduction:** After the age of 50, the incidence of osteoarthritis (OA) and risk of knee pain is greater in females, coinciding with the time of hormonal changes associated with menopause. It has previously been shown by others that female mice have reduced chondropathy compared to males after surgical joint destabilization. This study investigated the mechanisms behind this difference.

**Materials and Methods:** Surgical induction of OA was performed in C57BL6 and DBA1 males and females. Randomized groups received either sham, destabilization of the medial meniscus (DMM), or partial meniscectomy (PMX) surgery. Ovariectomy (OVX) was performed on a group of female C57BL6 mice prior to PMX surgery. Spontaneous pain‐like behaviour was evaluated by weight bearing assessments on a Linton incapacitance tester. Behavioural activity was assessed using the Laboratory Animal Behaviour Observation Registration and Analysis System (LABORAS) to compare the sexes in both the C57BL6 Naïve and PMX surgery groups. Whole knee joints were collected for either gene expression of pain‐associated molecules or to assess disease severity from histological sections following the Osteoarthritis Research Society International (OARSI) scoring system.

**Results:** We confirmed that, at both 8 and 12 weeks, in both strains, and following either destabilization surgery, females had reduced chondropathy compared to males. Despite this, male and female mice displayed the same onset and severity of spontaneous pain behaviour, by incapacitance testing, following joint destabilization (onset 11 weeks post‐DMM, 8 weeks post‐PMX). LABORAS activity was unable to distinguish changes in behaviour between sexes or surgical groups. Disease severity and pain behaviour was not affected by OVX prior to joint destabilization. At the time of onset of pain, female mice, but not male mice, displayed upregulation of genes encoding Gdnf, Nrtn, Ntf3 and Ntf5, and Pspn was significantly downregulated.

**Discussion:** Despite reduced chondropathy, young females demonstrated the same onset of pain‐like behaviour compared to males suggesting females have heightened pain sensitivity in OA. This does not appear to be due to sex differences in behavioural activity. Regulation of a distinct set of pain‐associated molecules in female joints at the time of pain onset may identify novel putative sex‐dependent pain targets.

### *FHL2* promoter DNA methylation increases with chronological age in joint tissues and could impact target gene expression

R. C. Fulea ^*^; D. A. Young^*^; G. Bou‐Gharios^†^; L. N. Reynard^*^

^*^Newcastle University Biosciences Institute, Newcastle upon Tyne, NE1 3BZ; ^†^Institute of Ageing and Chronic Disease, University of Liverpool, Liverpool, L7 8TX

**Introduction:** Genome‐wide DNA methylation analysis of 179 cartilage samples revealed 716 CpG sites that show age‐related changes, majority located in gene promoters. The effect of these changes on gene expression is unknown, although DNA methylation is usually associated with decreased target expression. The aims of this study were to validate several of these CpGs within the *FHL2* gene promoter in joint tissues and to determine what effect they have on *FHL2* expression.

**Materials and Methods:** Knee cartilage, synovium and fat pad from osteoarthritis patients were acquired from the Newcastle Biobank. DNA methylation was assessed using bisulphite pyrosequencing. The regions encompassing the CpGs were cloned in a pCpGL‐basic‐luciferase vector, in vitro methylated and transfected into SW1353 chondrosarcoma cells to analyse the regulatory activity.

**Results:** Three *FHL2* CpGs identified in the initial study and six new neighbouring CpGs showed increased DNA methylation with increasing age in a new cohort of cartilage samples (*p* < 0.0001, *n* = 72). These changes were observed also in synovium and fat pad tissues (*p* < 0.005, *n* = 22 per tissue). The activity of the *FHL2* promoter, showed seven fold increase in reporter activity compared to the promoter free control (*p* < 0.0001). Upon in vitro methylation of the promoter region, reporter activity was 3.5× lower than when the region was unmethylated (*p* < 0.0001).

**Discussion:** The CpGs in *FHL2* promoter region show age‐associated changes in cartilage, synovium and fat pad tissue. The luciferase assay showed that this region can function as a promoter and moreover this activity is inhibited by DNA methylation. These changes suggest that the expression of *FHL2* can be impacted by ageing and therefore could give insight into a mechanism of joint ageing.

### Engineering the gradient extracellular matrix in osteochondral tissue

J. P. K. Armstrong; C. Li; M. M. Stevens

*Department of Materials, Department of Bioengineering, and Institute of Biomedical Engineering, Imperial College London, London, UK*

**Introduction:** The osteochondral interface between cartilage and bone enables effective transmission of stresses during articulation of joints. This is enabled by transitions in the composition and organization of the extracellular matrix, structural features that develop under the influence of morphogen gradients. Here, I will introduce our benchtop approach enabling biomaterials to be encoded with morphogen gradients to direct matrix assembly during osteochondral tissue engineering.

**Materials and Methods:** Density‐driven phase separation was achieved by injecting a hydrogel precursor at a defined rate into another base solution of hydrogel precursor containing a density modifier. For osteochondral tissue engineering, both solutions contained gelatin methacryloyl (GelMA), human mesenchymal stem cells and photoinitiator, while the base solution contained Ficoll as a density modifier and the injection solution contained bone morphogenetic protein 2 (BMP2) sequestered in heparin methacrylate (HepMA). After the gradient was established, the system was photocrosslinked and cultured for 28 d in osteochondral differentiation medium.

**Results:** We used density‐driven phase separation to generate tunable fluid gradients that were then encapsulated by gelation or polymerization. This method, requiring only a mould and a micropipette injector, was used to encode a range of gradients into common biomaterial systems. Using this approach, we cast gradients of BMP2 into GelMA hydrogels containing human mesenchymal stem cells. Osteochondral tissues were generated over 28 d, with the sustained release of BMP2 triggering local osteogenesis and mineralization at one end of the cartilaginous tissue construct, as evidenced by histology and Raman spectroscopy.

**Discussion:** The use of a fluid redistribution strategy ensured the formation of a tissue with highly integrated zonal matrix, avoiding common issues with delamination or the exclusion of cells at the tissue interface. As well as potential suitability as a tissue graft, this system provided insight into the formation of the osteochondral tidemark, suggesting potential applications for modelling tissue development. Moreover, the simplicity, accessibility and versatility of this gradient‐casting platform lends itself to widespread application for the engineering other gradient tissues or heterogeneous culture systems.

### Inter‐alpha‐inhibitor supports in vitro expansion of both primed and naïve human pluripotent stem cells in defined, xeno‐free conditions

S. Pijuan‐Galito^*,†^; J. L. Thompson^†^; L. C. Lewis^†^; C. Tamm^‡^; C. Annerén^‡^; C. Denning^†^; C. L. R. Merry^†^

^*^School of Pharmacy University of Nottingham, Nottingham, UK; ^†^School of Medicine University of Nottingham, Nottingham, UK; ^†^Institute for Medical Biochemistry and Microbiology, Uppsala University, Uppsala, Sweden

**Introduction:** Human pluripotent stem cells (hPSCs), are a promising tool in cell biology. Recently, human naïve pluripotent cells (hNPSCs) have been reported, showing a biomolecular profile closer to the pre‐implantation embryo and increased differentiation potential. The derivation of hNPSCs is still reliant on feeder cells and hypoxic conditions. We have shown that human serum‐derived Inter‐a‐inhibitor (IαI) can support the long‐term expansion of hPSCs in vitro using coating‐free conditions. We now show that IαI supports transition and expansion of hPSCs into the newly discovered naïve pluripotency in feeder‐free, normoxic conditions.

**Materials and Methods:** hPSCs cultured on IαI were transitioned to hNPSCs using modified RSeT and 2iGöY formulations in normoxic, feeder‐free and coating‐free conditions. Western blotting, qPCR and high‐resolution confocal microscopy was performed to investigate the hNPSCs biomolecular profile. Seahorse X96 Mito‐stress assay was performed to measure oxidative respiration.

**Results:** The hNPSCs obtained in IαI conditions show typical dome morphology, increased expression of naïve markers and relevant intracellular signalling. Integrin profile shows reduced total integrin protein content as well as reduced FAK phosphorylation. DNA hypomethylation was confirmed using 5‐mC staining coupled with confocal imaging. Seahorse assay was performed to confirm switch from glycolysis to oxidative phosphorylation. Finally, hNPSCs were transferred to a chemically‐defined hydrogel set‐up (peptide‐based, FEFEFKFK), with confocal microscopy showing naïve colonies growing into large 3D clusters expressing naïve markers KLF17, DPPA3 and SSEA‐4.

**Discussion:** We present a defined and feeder‐free method for naïve and primed pluripotent stem cells. The culture method is flexible to biomaterial formats and hydrogel technologies, as shown by the easy adaptation to a defined, naked hydrogel. This new culture method has the potential to widen the use of hNPSCs in basic research and in combination with tissue engineering for early development modelling and cell therapy strategies.

### TNF alpha‐stimulated gene 6 (TSG‐6) is weakly chondroprotective in murine OA but does not account for FGF2‐mediated joint protection

L. Zhu ^*,1^; S. Donhou^*,1^; A. Burleigh^*^; J. M. Zarebska^*^; M. Curtinha^*^; I. Parisi^*^; S. N. Khan^*^; F. Dell’Accio^†^; A. Chanalaris^*^; T. L. Vincent^*^

^*^Arthritis Research UK Centre for Osteoarthritis Pathogenesis, Kennedy Institute of Rheumatology, University of Oxford, Oxford, United Kingdom; ^†^William Harvey Research Institute, Queen Mary, University of London, London, United Kingdom

**Introduction:** Tumour necrosis factor alpha‐stimulated gene 6 (TSG‐6) is expressed in response to a range of pro‐inflammatory mediators and has purported tissue‐protective and anti‐inflammatory effects. TSG‐6 has been detected at high levels in the synovial fluid of patients with rheumatoid arthritis and osteoarthritis. We have previously shown that TSG‐6 is regulated in the mouse joint following surgical destabilization of the medial meniscus (DMM), in a highly mechanosensitive and fibroblast growth factor 2 (FGF2)‐dependent manner. As FGF2^‐/‐^ mice develop accelerated OA, and injury‐induced FGF2‐dependent genes were downregulated in TSG‐6^‐/‐^ mouse joints, we speculated that the chondroprotective role of FGF2 may be mediated through TSG‐6.

**Materials and Methods:** 42 genes were quantified by real‐time PCR in whole joints post DMM (6h and 7 days). Joint pathology was assessed in male and female FGF2^‐/‐^, TSG‐6^‐/‐^, TSG‐6^tg^ (overexpressing), FGF2^‐/‐^;TSG‐6^tg^ (8 weeks only) mice with their respective controls at 8 and 12 weeks following DMM. Cartilage repair was tested 8 weeks following focal cartilage injury in TSG‐6^tg^ and control mice. FGF2 release after cartilage injury was measured by V‐PLEX bFGF kit.

**Results:** 15 inflammatory genes were significantly upregulated in TSG‐6^‐/‐^ joints post DMM including *IL1α*, *Ccl2* and *Adamts5* compared with wild type. Six genes were significantly suppressed in TSG‐6^‐/‐^ joints including *Timp1*, *Inhibin βA* and *podoplanin*. Accelerated OA was seen at 12 weeks post DMM in male TSG‐6^‐/‐^ mice and a reciprocal improvement in disease was seen in TSG‐6^tg^ mice. There was no significant difference observed in cartilage repair between genotypes. FGF2 release in the conditioned medium after injury was not influenced by genotype. TSG‐6 overexpression was unable to prevent accelerated OA in FGF2^‐/‐^ mice.

**Discussion:** TSG‐6 influences early gene regulation in the destabilized joint and is weakly chondroprotective in murine OA. Although strongly FGF2‐dependent, TSG‐6 does not account for FGF2‐mediated joint protection.

### D‐Net: An AI model for aligning volumes, developed for cartilage segmentation from CT scans contrasted with a type II collagen binding peptide containing di‐iodotyrosine

J. Q. Zheng ^*^; B. W. Papiez^†^; N. H. Lim^*^

^*^The Kennedy Institute of Rheumatology, University of Oxford, United Kingdom; ^†^Big Data Institute, University of Oxford, United Kingdom

**Introduction:** Di‐Iodotyrosinated Peptide Imaging of Cartilage (DIPIC) by computed tomography (CT) uses a type II collagen targeted radio‐contrast agent to visualize cartilage by X‐rays. Manual segmentation of cartilage from the contrast‐enhanced images is time consuming, while semi‐automated methods based on X‐ray absorption only reliably segments out cartilage in the weight bearing region. We hypothesize subtraction of non‐contrasted CT scan from DIPIC scans would yield better segmentation of cartilage, but this requires accurate alignment of both scans. We explore the use of Artificial Intelligence (AI) models for this alignment task.

**Materials and Methods:** The CT scans of mouse tibiae were obtained with and without contrast. The nature of the animal scanner meant that no standardization of pose between scans was possible. Thus, in addition to being rotated within the field of view, the two scans may be to be rotated in any orientation compared to each other. We compared three previously published AI methods and two translational models and found that they were unable to cope with the full range of rotation. To address this, we developed D‐Net. The networks were trained with 50 CT volumes, synthetically translated and rotated. The original 512x512x512 with resolution 10^3^ μm^3^/vox scan was subsampled to 64x64x64 (80^3^ μm^3^/vox) before fed into the network due to memory limitations. Testing was on another 50 CT volumes unseen by the network and on paired volumes with and without contrast. We report the difference between the predicted and expected translation (TE), rotation (RE).

**Results:** D‐Net significantly outperformed all other networks, achieving a TE of 70 μm (sub‐voxel) and a RE 4°.

**Discussion:** While D‐Net performed well on the down‐sampled volumes, it was impractical to train it on the original sized data. Further development is required to align the volumes at the original resolution. We postulate however, that trained on the appropriate dataset, D‐Net would be able to perform alignment of any 2 volumes from any rotation. Additionally, the calculations for this to occur takes less than a second, enabling real‐time applications.

